# Oleylamine Aging of PtNi Nanoparticles Giving Enhanced Functionality for the Oxygen Reduction Reaction

**DOI:** 10.1021/acs.nanolett.1c00706

**Published:** 2021-04-26

**Authors:** Gerard M. Leteba, Yi-Chi Wang, Thomas J. A. Slater, Rongsheng Cai, Conor Byrne, Christopher P. Race, David R. G. Mitchell, Pieter B. J. Levecque, Neil P. Young, Stuart M. Holmes, Alex Walton, Angus I. Kirkland, Sarah J. Haigh, Candace I. Lang

**Affiliations:** † Catalysis Institute, Department of Chemical Engineering, University of Cape Town, Corner of Madiba Circle and South Lane, Rondebosch 7701, South Africa; ‡ Department of Materials, University of Manchester, Manchester M13 9PL, United Kingdom; § Electron Physical Sciences Imaging Centre, Diamond Light Source Ltd., Oxfordshire OX11 0DE, United Kingdom; ∥ Electron Microscopy Centre, Innovation Campus, University of Wollongong, Wollongong, New South Wales 2517, Australia; ⊥ Department of Materials, University of Oxford, Parks Road, Oxford OX1 3PH, United Kingdom; # School of Engineering, Macquarie University, Sydney, New South Wales 2109 Australia; ∇ Beijing Institute of Nanoenergy and Nanosystems, Chinese Academy of Sciences, Beijing, 101400, China; ○ School of Nanoscience and Technology, University of Chinese Academy of Sciences, Beijing, 100049, China; ◆ Department of Chemistry, University of Manchester, Manchester M13 9PL, United Kingdom; ¶ Department of Chemical Engineering and Analytical Science, University of Manchester, Manchester M13 9PL, United Kingdom; △ Photon Science Institute, University of Manchester, Manchester M13 9PL, United Kingdom

**Keywords:** ORR, electrocatalyst, nanoparticle, electron tomography, STEM-EDS, PEMFC

## Abstract

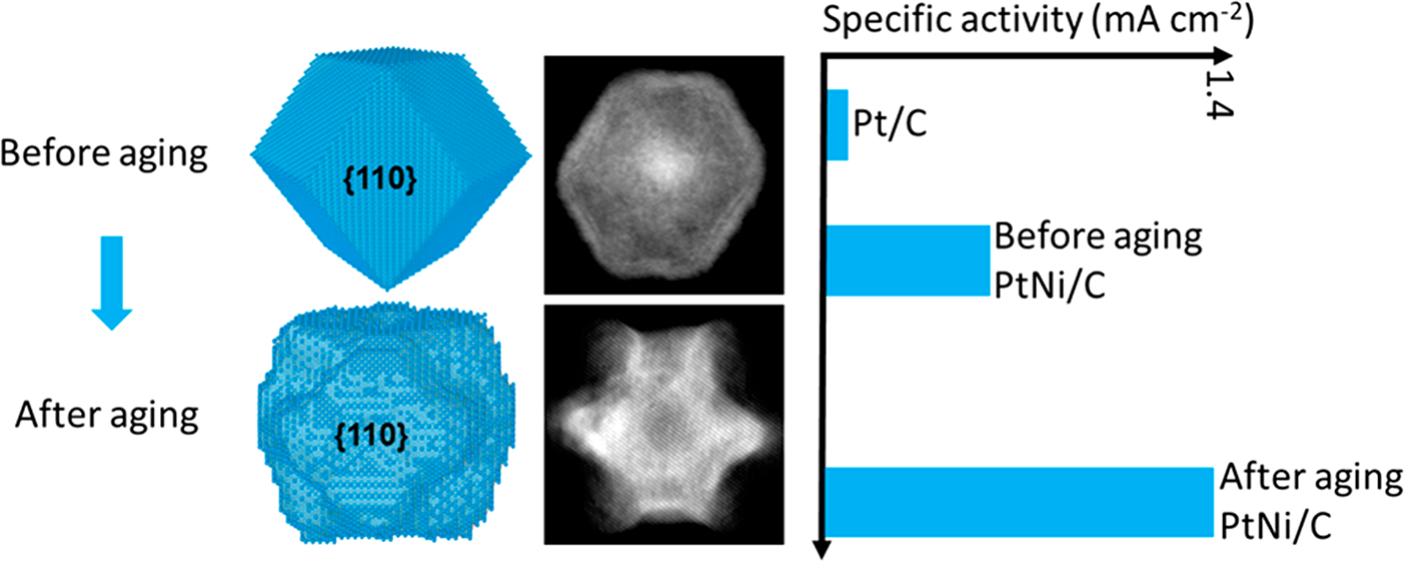

We report a rapid solution-phase strategy to synthesize alloyed PtNi nanoparticles which demonstrate outstanding functionality for the oxygen reduction reaction (ORR). This one-pot coreduction colloidal synthesis results in a monodisperse population of single-crystal nanoparticles of rhombic dodecahedral morphology with Pt-enriched edges and compositions close to Pt_1_Ni_2_. We use nanoscale 3D compositional analysis to reveal for the first time that oleylamine (OAm)-aging of the rhombic dodecahedral Pt_1_Ni_2_ particles results in Ni leaching from surface facets, producing aged particles with concave faceting, an exceptionally high surface area, and a composition of Pt_2_Ni_1_. We show that the modified atomic nanostructures catalytically outperform the original PtNi rhombic dodecahedral particles by more than two-fold and also yield improved cycling durability. Their functionality for the ORR far exceeds commercially available Pt/C nanoparticle electrocatalysts, both in terms of mass-specific activities (up to a 25-fold increase) and intrinsic area-specific activities (up to a 27-fold increase).

Commercial carbon-supported platinum nanoparticles (Pt/C) are effective electrocatalysts for both the anodic hydrogen oxidation reaction (HOR, 4H_2_ → 4H^+^ + 4e^–^) and the cathodic oxygen reduction reaction (ORR, O_2_ + 4H^+^ + 4e^–^ → 2H_2_O) in polymer electrolyte membrane fuel cells (PEMFCs).^1,2^ The commercial viability of PEMFCs is, however, limited by the high cost of Pt and the sluggish kinetics of the ORR.^1,3,4^ Research has shown that alloying Pt with 3d transition metals enhances catalytic functionality while reducing the Pt load with potential cost savings.^2,3^ Substantial efforts have therefore been directed at novel solution-phase synthesis methods for Pt-based nanoalloys, which can exhibit enhanced catalytic activity as a result of the surface structure and chemistry.^2,5−7^ Li et al. have shown that dealloyed Pt–Ni nanowires exhibit massively enhanced mass activity (52 times improvement versus commercial Pt/C),^8^ which is attributed to the formation of ultrafine jagged nanowires with highly stressed, undercoordinated surface configurations. Up to now, this outperforms most of the Pt–Ni particle-based catalysts. Open-framework Pt_3_Ni nanostructures have been found to display enhanced functionality as a result of their high surface area, high-index surface facets, and Pt enrichment at surface sites,^5^ whereas introducing rougher or concave topography to Pt–Ni nanoparticles can provide a conservative improvement in ORR activities.^9,10^ Crystal faceting is another important factor to optimize ORR catalytic activities. It is found that anisotropic morphologies bounded by {111} crystal planes, display enhanced catalytic performance compared to the lower-index ({100} and {110} bounded) surfaces due to the favorable adsorption of hydroxyl rather than oxygen on high-index surfaces.^11−13^ However, all of the available nanoparticle systems for ORR have limitations associated with the complexity of the synthesis route, their cycling stability, or reduced catalytic function after prolonged operation.

The scalability and controllability of wet chemistry synthesis makes it an attractive route for developing alloyed nanocatalysts with good control of surface morphology.^5,14,15^ The nucleation and growth of metallic nanostructures from solution is governed by kinetic growth parameters as well as by the nature and strength of surfactants, local concentration, temperature, and so forth.^15−17^ Particularly for bimetallic systems, the shape, size, and composition of the product can be highly sensitive to the exact synthesis conditions, thus a robust synthetic route is highly desirable for subsequent scale up.^11,15,16^

Here, we report a facile, scalable, and robust synthetic procedure for the preparation of monodisperse PtNi nanoalloys with controllable morphology and surface composition. This novel route uses tetrabutylammonium borohydride (TBAB) as the reductant in a mixture of hydrophobic surfactants, oleylamine (OAm), and octadecylamine (ODA), with oleic acid (OLEA) or trioctylamine (TOA) as the third surfactant component, and results in PtNi nanoparticles with a predominantly rhombic dodecahedral morphology. Aging in OAm is then performed where OAm is added as a surfactant to deflocculate the aggregated nanoparticles and improve their processability by creating a homogeneous dispersion. We measured the ORR performance of our as-synthesized PtNi nanoparticles and the OAm-aged PtNi nanoparticles and found that both exhibit significantly enhanced ORR functionality and improved stability compared with commercially available Pt/C electrocatalysts with ORR functionality values exceeding the DoE target.^8^ We found that our OAm-aged PtNi nanoparticles catalytically outperformed the as-synthesized PtNi nanoparticles so we used an accurate 3D atomic model of the nanoparticles based on high-angle annular dark field scanning transmission electron microscopy (HAADF-STEM) single particle reconstruction and energy dispersive X-ray spectroscopy (EDXS) to understand the changes in morphology and elemental distribution due to aging. We found that OAm aging results in preferential etching to produce Pt rich, concave surfaces by leaching of Ni, which is likely responsible for the improved performance.

PtNi nanostructures were successfully solution-grown via a simultaneous reduction of nickel(II) acetate tetrahydrate and chloroplatinic acid solution (precursor salts), using TBAB as the reductant. The synthesis medium was a ternary mixture of hydrophobic surfactants OAm, ODA, and OLEA in a high boiling point solvent 1-octadecene (1-OD). The effect of the surfactant OLEA was investigated by replacing OLEA with TOA in this synthesis protocol. Particles synthesized with the OLEA surfactant are referred to here as PtNi-OLEA-Solid while those synthesized with TOA are referred to as PtNi-TOA-Solid. After aging in OAm, the samples are referred to as PtNi-OLEA-Aged and PtNi-TOA-Aged for the OLEA synthesis and TOA synthesis, respectively. We note here that nucleation was slow, followed by rapid growth. Our capacity to monitor the growth mechanisms of these colloidal alloys was accordingly limited.

The two as-synthesized types of PtNi alloy nanoparticles (PtNi-OLEA-Solid and PtNi-TOA-Solid) are observed to have similar morphologies, consisting of smoothly faceted rhombic dodecahedra with predominantly {110} facets (Figure 1a–d for PtNi-OLEA-Solid and Figure 1a, e–g for PtNi-TOA-Solid). For both PtNi-OLEA-Solid and PtNi-TOA-Solid, high quality monodispersed nanoparticles are observed. Histograms showing the diameters of the as-synthesized nanoparticles, calculated from transmission electron microscope (TEM) images of 250–300 randomly selected individual nanoparticles, show a narrow average particle size distribution (means ± standard deviations) of PtNi-OLEA-Solid (17.3 ± 1.6 nm), PtNi-TOA-Solid (16.8 ± 1.3 nm), PtNi-OLEA-Aged (17.9 ± 1.5 nm), and PtNi-TOA-Aged (16.6 ± 1.5 nm) nanoparticles (Figure S1a–d), thus requiring no size-selective processing to achieve uniform particle size. Both TOA and OLEA protocols produced similar rhombic dodecahedral-shaped nanoparticles, demonstrating that substituting the OLEA and TOA had little influence on the final synthesized structure. We conclude that the procedure is robust to changes in surfactants and solvents, highlighting the scale up potential of this nanostructure synthesis route.

**Figure 1:**
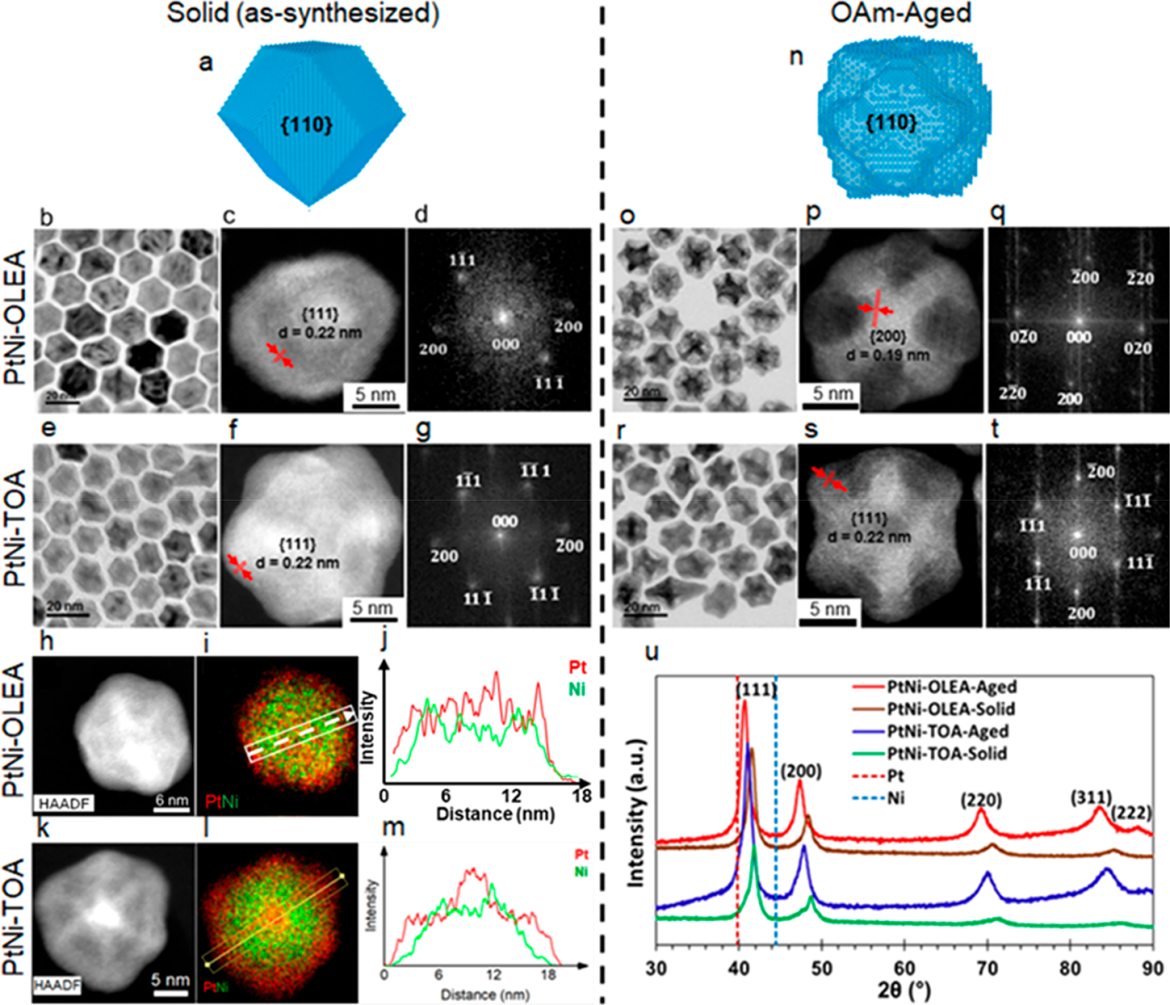
Structural characterization of PtNi nanoparticles. (a–d) and (a, e–g) Characterization of as-synthesized nanoparticle PtNi-OLEA-Solid and PtNi-TOA-Solid, respectively. (h–m) STEM–EDXS analysis of as-synthesized PtNi nanoparticles revealing the elemental distribution within single crystalline particles. (h,k) HAADF STEM images and (i,l) composite EDXS maps of Pt (red) and Ni (green) for PtNi-OLEA-Solid and PtNi-TOA-Solid, respectively. Line profiles extracted from the composite maps (i,l) are shown in (j,m) to illustrate the core–shell–shell PtNi nanoparticle structures. (n–q) and (n, r–t) Characterization of aged nanoparticles PtNi-OLEA-Aged and PtNi-TOA-Aged, respectively. (a,n) Schematic structures of these nanoparticles before and after OAm-aging. (b,e,o,r) TEM images of nanoparticle populations. (c,f,p,s) High-resolution HAADF-STEM images with measured lattice spacings and (d,g,q,t) are the corresponding fast Fourier transforms with indexed diffraction spots. (u) PXRD patterns of PtNi-OLEA and PtNi-TOA nanoparticles before and after aging showing fcc solid solutions of Pt and Ni. The positions of pure Pt(111) and Ni(111) peaks are indicated by dashed lines.

OAm serves to deflocculate the nanoparticles and also acts as an etchant to increase surface area. Aging as-synthesized nanoparticles in OAm for 3 weeks, we observe selective dissolution of nanoparticle surfaces during aging, creating concave facets in the rhombic dodecahedral structure although the overall size distribution is unaffected (Figure 1n–t and Figure S1).

Atomic resolution HAADF-STEM imaging of Solid and OAm-Aged nanoparticles revealed the internal crystal structure and geometry of these nanoparticles (Figure 1c,f,p,s). Fourier transforms (FTs) obtained from atomic resolution images of individual nanoparticles and selected area electron diffraction (SAED) reveal these are perfect single crystals with the expected face centered cubic (fcc) structure despite their unusual morphology (Figure 1d,g,q,t and Figure S1e,f). A lattice parameter of 0.38 ± 0.02 nm was measured for both PtNi-OLEA-Aged and PtNi-TOA-Aged samples via HAADF-STEM images (see Table S1).

Powder X-ray diffraction (PXRD) (Figure 1u) further confirmed a fcc phase solid solution of Pt and Ni (Table S1). Partial incorporation of smaller atoms like Ni into the Pt crystal lattice induces a shift to higher 2θ angles relative to pure Pt. This in turn facilitates ORR catalytic performance by creating a favorable surface platform to weaken oxygen binding energy or surface-adsorbed hydroxyl (OH) species on Pt.^19,20^ A simple analysis of the {111} diffraction peaks using Vegard’s rule gives the composition of the PtNi-OLEA-Solid and the PtNi-TOA-Solid particles as Pt_29_Ni_71_ and Pt_37_Ni_63_ respectively. The high-angle diffraction peaks become more prominent after OAm-aging, suggesting lower lattice strain and enhanced nanoparticle crystallinity compared to the as synthesized structures. Aging of the PtNi nanoparticles is observed to also result in a shift of the diffraction peaks toward lower angles, for example, the {111} diffraction peak of PtNi-TOA shifts from 2θ = 41.841° to 41.023°. This indicates that the interplanar spacings are increased on aging from 0.216 to 0.220 nm, which is in agreement with the change in *d*-spacing calculated from electron diffraction patterns (Table S1). The increasing *d*-spacing after aging is attributed to partial dissolution of Ni from the PtNi fcc lattice (Vegard analysis gives compositions of PtNi-OLEA-Aged and PtNi-TOA-Aged as Pt_54_Ni_46_ and Pt_49_Ni_51_).

Scherrer analysis of the PXRD data revealed average nanoparticle crystallite sizes of 19.4 ± 0.01 nm and 17.3 ± 0.01 nm for PtNi-OLEA-Aged and PtNi-TOA-Aged, respectively. Both of these values are slightly larger than the average values obtained from the TEM/STEM size analysis although they are within the standard deviation of both techniques.^21^

To further characterize the unusual nanoparticle morphology after etching, the averaged 3D structure of the nanoparticle population was determined by HAADF-STEM single particle reconstruction using about 400 PtNi-OLEA-Aged nanoparticles (Figure 2 and Figure S4; for details, see experimental methods and ref (22)). The reconstruction revealed a 3D morphology consisting of a rhombic dodecahedron with concave facets. The rhombic dodecahedron morphology was further confirmed to be representative of the wider population by comparison of the projected 3D structure with experimental images for different nanocrystals (Figure S4). A full 3D atomic model (Figure 2b,j) was determined by combining the reconstructed mean 3D structure and crystallographic information from the atomic resolution TEM images (Figure 2d,l). Comparing multislice image simulations (Figure 2c,e,f and k,m,n) with experimental atomic resolution TEM images of aged nanoparticles with different crystal orientations (Figure 2d,g,h and l,o,p) reveals a good qualitative match between the broad intensity distribution and atomic structure.

**Figure 2:**
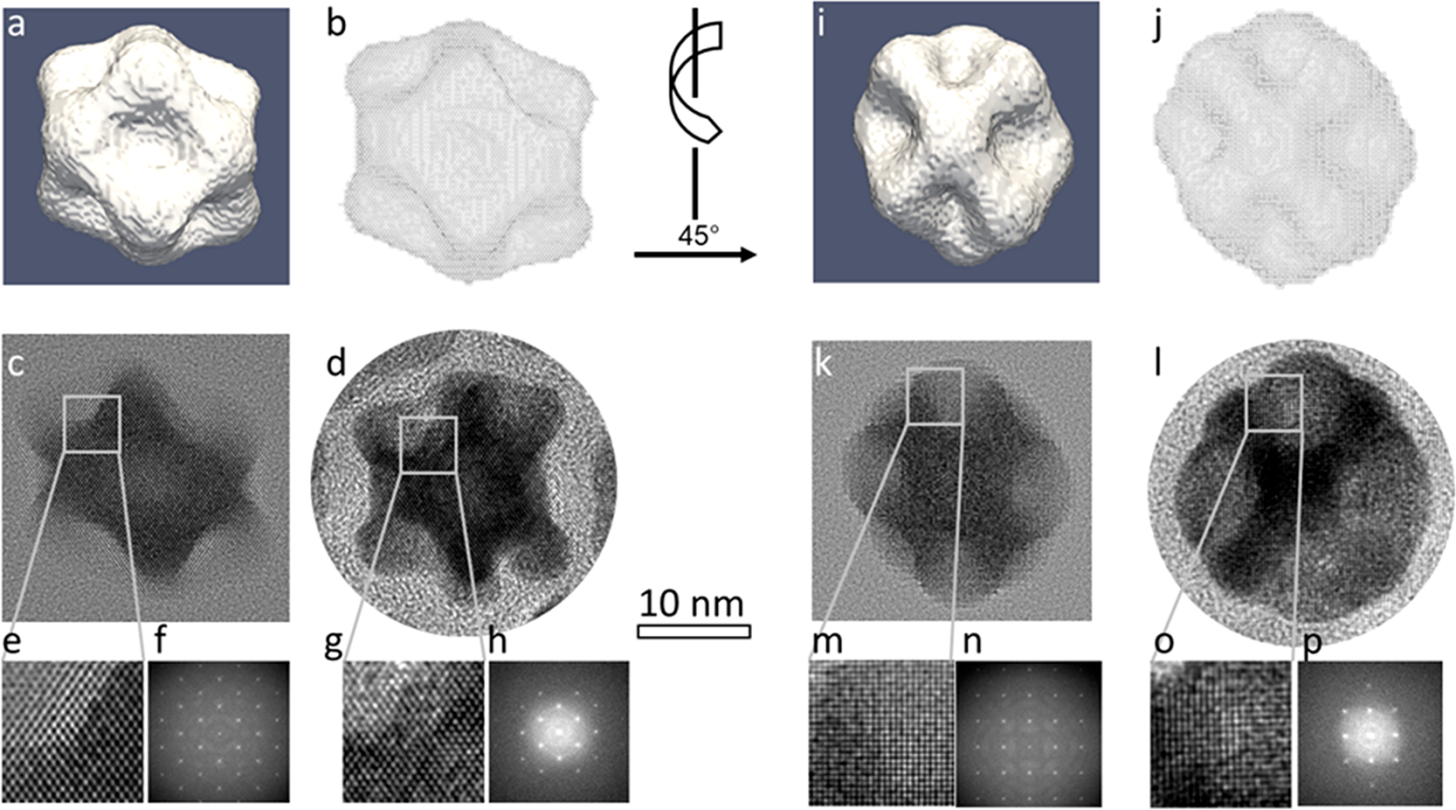
Comparison of the 3D atomic reconstruction with experimental TEM images for PtNi-OLEA-Aged nanoparticles, viewed along (a–h) a ⟨110⟩ zone-axis and (i–p) a ⟨100⟩ zone-axis. (a,i) Isosurface rendering of the 3D reconstruction representing the averaged morphology of PtNi-OLEA-Aged nanoparticles. (b,j) Visualization of the proposed atomic model of rhombic dodecahedral shape. (c,k) Simulated HRTEM images of the atomic model and (d, l) HRTEM images of an PtNi-OLEA-Aged nanoparticle. (e,m) Enlarged view of the HRTEM images for the atomic model in (c,k). (f,n) FT of the HRTEM images of the atomic model. (g,o) Enlarged view of the HRTEM images in (d,l). (h,p) FT of the real HRTEM images. Scale bars for (c,d,k,l) are 10 nm.

The composition and elemental distribution within these bimetallic nanoparticles before and after OAm-aging were then analyzed by EDXS (Figure 1h–m, Figure 3, Figure S3, and Figure S5). This showed the as-synthesized bimetallic nanocrystals to have mean compositions of Pt_32_Ni_68_ (PtNi-OLEA-Solid) and Pt_34_Ni_66_ (PtNi-TOA-Solid), close to Pt_1_Ni_2_ and consistent with the PXRD prediction. Subsequent to aging in OAm, the nanoparticles exhibited substantial reduction in their Ni content having average compositions of Pt_64_Ni_36_ (PtNi-OLEA-Aged) and Pt_56_Ni_44_ (PtNi-TOA-Aged). These are close to Pt_2_Ni_1_ providing further evidence of OAm induced Ni surface and subsurface dissolution during aging.^5^ Intensity profiles from the STEM–EDXS elemental maps reveal compositional inhomogeneity within individual nanoparticle structures (Figure 3e–g and l–n). The Pt-rich core could be the remnants of the Pt seed from nucleation at the initial coreduction.^23^ Note that this seed is usually close to the geometric center of the particle but can also be found to be off-center. The preferential segregation of Pt to the apexes or surfaces might be expected thermodynamically, as pure Pt has a larger lattice parameter than Ni so this segregation will reduce the total lattice strain of the system, consistent with the pure “Pt skin” reported previously in model surfaces.^3,13,19^ On the basis of atom column intensities and interatomic distances in atomic resolution HAADF images of an aged nanoparticle, the estimated Pt-rich skin is about 3–4 atom layers (Figure S7) consistent with X-ray photoelectron spectroscopy (XPS) analysis (Figure S8). More detailed 3D characterization via spectroscopic single particle reconstruction revealed that the aged PtNi nanoparticles possess concave Ni-rich surfaces and a Pt-rich outer frame, alongside the Pt-rich core.^22^

**Figure 3:**
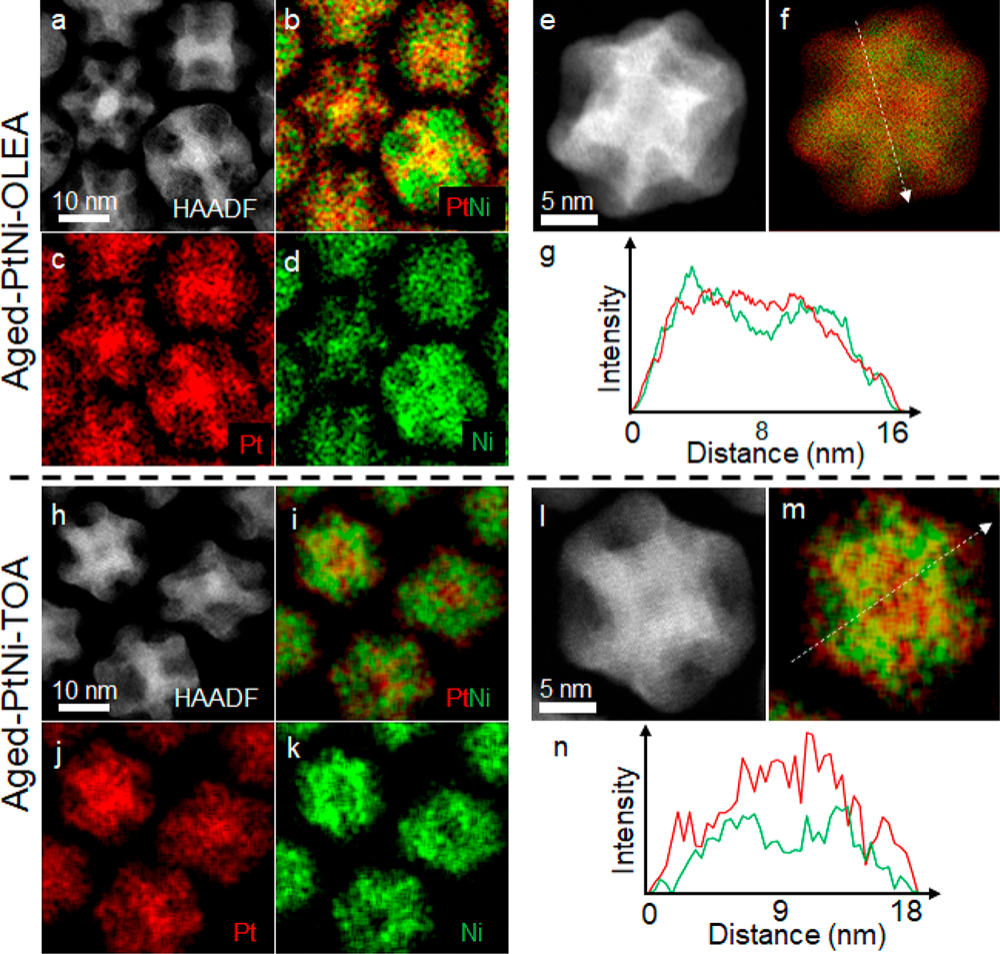
STEM–EDXS elemental distribution analysis of PtNi-OLEA (a–g) and PtNi-TOA (h–n) nanoparticles after OAm-aging. HAADF STEM images (a,e,h,l) are shown alongside STEM EDXS elemental maps for Pt (c,j) and Ni (d,k) in the same specimen region. Composite elemental maps demonstrate the relative locations of Pt and Ni (b,f,i,m). Line profiles extracted at the positions shown in the composite maps (f,m) are shown in (g,n) to illustrate the inhomogeneous spatial distributions of Pt and Ni within individual nanoparticle structures, as well as the Pt-enriched cores.

The nanoparticle’s monodispersity, high surface area and Pt-rich surface atop a PtNi subsurface is highly promising for their catalytic performance. To test the catalytic activity, four samples (PtNi-OLEA-Solid, PtNi-TOA-Solid, PtNi-OLEA-Aged, and PtNi-TOA-Aged) were dispersed on highly conductive, high surface area carbon supports (Vulcan XC-72R), via a colloidal-deposition method (see Supporting Information Methods for details). These carbon supported samples are referred to as PtNi-OLEA-Solid/C, PtNi-TOA-Solid/C, PtNi-OLEA-Aged/C, and PtNi-TOA-Aged/C. Bright-field (BF) TEM images of Solid-samples (Figure S9a,b) and Aged-samples (Figure 4a,b) show their good dispersion with no apparent alteration in surface structure and particle size distribution. This allowed electrochemical investigations of their true catalytic specific activities.

**Figure 4:**
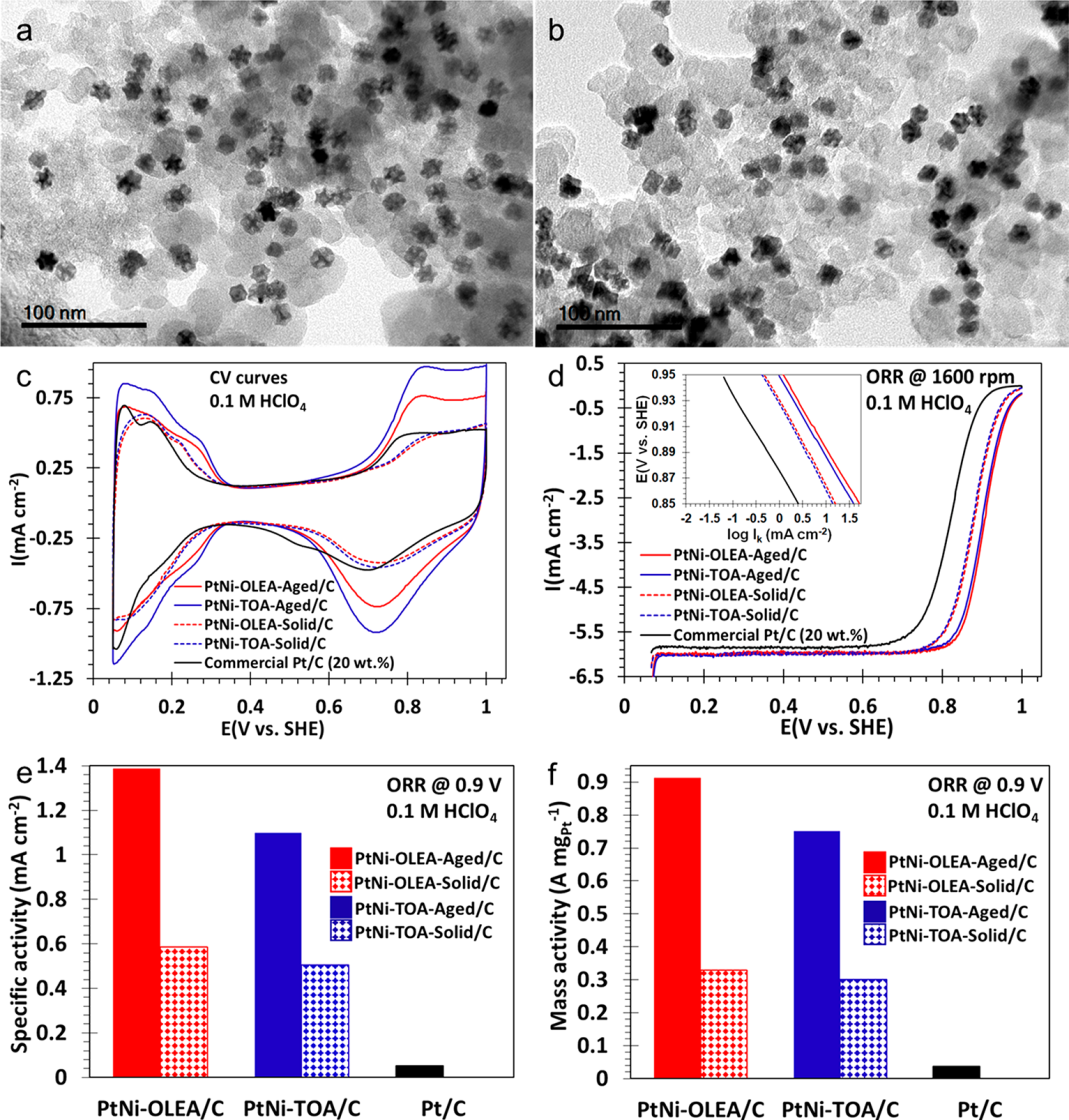
BF-TEM images of (a) PtNi-OLEA-Aged/C and (b) PtNi-TOA-Aged/C nanoparticles. (c) Cyclic voltammograms of activated binary PtNi-OLEA-Aged/C (red, solid plot), PtNi-OLEA-Solid/C (red, dashed plot) PtNi-TOA-Aged/C (blue, solid plot), PtNi-TOA-Solid/C (blue, dashed plot), and commercial Pt/C (black), (d) ORR polarization curves of samples before and after OAm aging (insert is the corresponding Tafel plots), (e) intrinsic area-specific activities, and (f) mass-specific activities at +0.90 V (vs SHE) after 30 potential cycles of activation of electrocatalysts.

Both cyclic voltammograms (CV) (Figure 4c) and CO stripping curves (Figure S10b, solid lines) were used to estimate the electrochemically active surface area (ECSA) of the synthesized nanocatalysts (20 wt % loading) and the commercial Pt/C electrocatalyst (Alfa Aesar, HiSpec, 20 wt % loading) (see Supporting Information Methods for details). The CV curves show the hydrogen adsorption/desorption (∼0.05–0.35 V vs standard hydrogen electrode, (SHE)) and oxide formation/reduction (∼0.70–1.00 V vs SHE) curves. Peak currents for these nanostructures were PtNi-TOA-Aged/C > PtNi-OLEA-Aged/C ≫ commercial Pt/C. Both ECSA_Hupd_ (Hupd = under-potentially deposited hydrogen) and ECSA_CO_ were calculated via normalization of the measured charges *Q*_H_ (hydrogen adsorption) and CO_ads_ (CO adsorption), adsorbed on the electrocatalysts using 210 μC/cm^25^ and 420 μC/cm,^26^ respectively. The ECSA_Hupd_ scaled as commercial Pt/C (70.9 m^2^ g_Pt_^–1^) > PtNi-TOA-Aged (68.1 m^2^ g_Pt_^–1^) > PtNi-OLEA-Aged (65.7 m^2^ g_Pt_^–1^) whereas the ECSA_CO_ scaled as commercial Pt/C (87.2 m^2^ g_Pt_^–1^) > PtNi-TOA-Aged (74.7 m^2^ g_Pt_^–1^) > PtNi-OLEA-Aged/C (71.7 m^2^ g_Pt_^–1^) (Table S2).

The good ECSAs of these binary alloy nanoparticles are believed to be associated with the high concentration of surface defects such as hollow edges/corners associated with the complex 3D morphology demonstrated in Figure 2. In addition, electrochemical Ni dissolution (dealloying) during activation may have created nanoporous surfaces, further increasing the surface area of these nanostructures.^9,27,28^ The ECSA_CO_/ECSA_Hupd_ ratios for these binary alloy nanoparticles were ∼1.1, indicative of nominal differences in terms of both H_ads_ and CO_ads_ surface coverage. All of the CO-stripping oxidation peaks for these nanostructures are located between +0.55 and +0.75 V (vs SHE). The presence of incorporated Ni just beneath the Pt surface resulted in the CO stripping peaks shifting to a lower potential than the commercial Pt/C electrocatalysts (between +0.6 and +0.85 V vs SHE) (Figure 4d), suggesting that the presence of Ni incorporated into Pt surfaces improves CO tolerance through Pt electronic modification and weakening of the Pt-CO bond^29,30^ for both PtNi-OLEA-Aged/C and PtNi-TOA-Aged/C.

The ORR polarization curves show two regimes: (a) the mixed kinetic-diffusion controlled-region (the true measure of the catalyst functionality) in the region between +0.85 and +1.00 V (vs SHE) and (b) the diffusion limited current density regime from ∼0.10 to ∼0.85 V (vs SHE) (Figure 4d). All of the polarization curves reached the diffusion limited-current density at ∼6.0 mA cm^–2^ (geometric) for ORR on all four PtNi nanostructures (Figure 4d, consistent with reported theoretical values (∼5.8–6.02 mA cm^–2^).^4^ This underlines the negligible influences of mass transport diffusion of molecular O_2_ to the working electrode as a result of the homogeneity and thinness of the tested films. Inset Tafel plots (Figure 4d obtained from the potentials in the range of +0.85–0.95 V (vs SHE), exhibit the following activity trend: PtNi-OLEA-Aged/C > PtNi-TOA-Aged/C > PtNi-OLEA-Solid/C > PtNi-TOA-Solid/C ≫ commercial Pt/C, indicating the superior catalytic performance of these synthesized binary nanoparticles. The mass activities and specific activity at +0.90 V (vs SHE) were obtained by normalizing the kinetic currents (*I*_k_) with the ECSA_Hupd_ and the Pt catalyst mass immobilized on the electrode, respectively. The kinetic current (*I*_k_) can be calculated using the Koutecky–Levich eq (Supporting Information Methods).^4^

The intrinsic area-specific activities (Figure 4e) of PtNi-OLEA-Aged/C and PtNi-TOA-Aged/C display ∼27-times (1.39 mA cm^–2^) and ∼21-times (1.10 mA cm^–2^) activity improvement, respectively, compared with the commercial Pt/C electrocatalyst (0.052 mA cm^–2^). The Pt mass-specific activities of PtNi-OLEA-Aged/C and PtNi-TOA-Aged/C exhibit ∼25-times (0.91 A/mg_Pt_) and 20-times (0.75 A/mg_Pt_) ORR enhanced functionality, respectively, compared with the commercial Pt/C electrocatalyst (0.037 A/mg_Pt_) (Figure 4f). We associate the higher activity of the PtNi-OLEA-Aged/C electrocatalyst with its more concave nanoparticle morphology, relative to the less concave PtNi-TOA-Aged/C electrocatalyst, resulting in greater surface area. The atomic surface sites on the concave surfaces of the PtNi-OLEA-Aged sample may also contribute to higher activity; previous studies have shown that electrocatalyst nanoparticles with stepped and terraced surfaces show improved catalytic activity when compared to nanoparticles with flat surfaces.^9,10^

The ORR area-specific activities of as-synthesized structures showed an increase of ∼11 times (PtNi-OLEA-Solid/C, 0.59 mA cm^–2^) and ∼10 times (PtNi-TOA-Solid/C, 0.51 mA cm^–2^) with respect to the commercial Pt/C electrocatalyst. The ORR mass-specific activities observed for PtNi-OLEA-Solid/C (0.33 A/mg_Pt_) and PtNi-TOA-Solid/C (0.30 A/mg_Pt_) nanostructures showed a slightly smaller increase of ∼9-times and ∼8-times higher than the commercial Pt/C, respectively. The OAm-aged nanostructures displayed an ∼2-fold increase in terms of mass-specific activity and an ∼3-fold enhancement of area-specific activities compared to the nanoalloys before aging. In addition, compared to the PtNi nanoframes by Chen et al.,^5^ the PtNi-OLEA-Aged/C here (regarded as an intermediate morphology between nanoframes and the faceted rhombic dodecahedrons) shows a slightly greater improvement in mass activity (25-fold increase versus their 22-fold increase) and area specific activities (27-fold increase versus their 16-fold increase), where both are compared to the Pt/C references. These activity enhancements highlight the exigent need to exploit fine chemical surface etching techniques for the creation of next-generation advanced hybrid nanocatalysts for catalytic reactions with uniquely uncoordinated Pt atomic surface arrangements. Table S3 compares the ORR activities for PtNi and other Pt alloys nanocatalysts from this and previous work. Although our system cannot currently compete with the best nanostructured PtNi catalysts: elaborately made with exotic morphologies consisting of jagged nanowires or nanoframes, the catalytic data presented here shows that these relatively solid PtNi particles (with ∼2 times larger diameters) can achieve competitive specific activities compared with most PtNi nanoparticle catalysts by introduction of concave surfaces.

The development of durable electrocatalysts has been the focus of much recent research.^5,31−33^ Durability testing of our nanoalloys over 5000 cycles (see Supporting Information Methods for details) showed that the ECSAs_Hupd_ decreased, relative to their initial value, by 40% for PtNi-OLEA-Aged/C and 36% for PtNi-TOA-Aged/C (Figure S11 and Table S2). In addition, CO stripping curves display a drop in current peaks (ECSA_CO_ loss of 40% for PtNi-OLEA-Aged/C and 33% for PtNi-TOA-Aged/C) and positive potential shift (from lower to higher) (Figure S10 and Table S2). All of these observations suggest selective Ni atomic surface and near-surface dissolution from the bulk alloys, resulting in diminished electrocatalytic performance and CO tolerance of the dealloyed nanoparticles. There was also substantial ORR functionality deterioration (Figure S10). These deteriorations in ECSA_Hupd_, ECSA_CO,_ and overall catalytic activities of these binary nanostructures during prolonged potential cycling could arise from a number of factors including electrochemical Ni dissolution, particle surface migration on the carbon support followed by coalescence/Ostwald ripening growth mechanism, or metal alloy oxide or hydroxide formation as a result of prolonged potential cycling and morphological deformations.^31,35^ STEM–EDXS analysis revealed substantial changes in the nanoparticle size, morphological deformation, and atomic rearrangements with particles becoming more spherical although Pt-enrichment is still apparent on the particle surface after cycling (Figure S12). In addition, more metal oxides exposed on the electrocatalyst’s surface may suppress the adsorption or diffusion of molecular O_2_ species to the working electrode interface and thus hamper ORR performance.

These results suggest that the higher ORR functionality of the fresh surface-aged PtNi alloy nanostructures is due to the newly evolved crystal facets formed via preferential OAm-Ni surface detachment. This is predicted to be further enhanced by electrochemical dealloying during the activation mechanism,^39^ thus altering the surface electronic properties and creating novel Ni subsurface distribution/rearrangement within the Pt nanostructures. In addition, surface defects such as hollows/corners, interfaces, high density of atomic steps and kinks, as well as distinct crystallographic planes, are believed to offer improved accessibility of reacting molecules to the catalyst surface active sites.^5,10,27,40^

In conclusion, the facile and robust solution-based synthetic approach reported here resulted in the formation of highly monodisperse, crystalline PtNi alloyed nanoparticles with composition of ∼Pt_1_Ni_2_. Aging in the surfactant OAm stabilized the structure and aged the surface of the nanoparticles, selectively dissolving Ni from the faces of the nanoparticles. This resulted in the formation of concave surfaces and many Pt-rich apexes, giving a mean nanoalloy composition close to Pt_2_Ni_1_. The surface-aged rhombic dodecahedral nanoparticles exhibited excellent electrocatalytic activity in the ORR, with PtNi mass-specific activities up to 25 times greater than a commercial Pt/C electrocatalyst. Preliminary durability measurements showed that mass-specific activity decayed, but only to a value which is still double the initial value for a commercial Pt/C electrocatalyst.

## Supporting Information Available

Experimental methods for characterization and electrochemical measurements, further TEM, STEM, EDS, XPS, FT-IR, and CV characterization data, as well as a comparison of the current work to recent ORR activities in the literature (PDF)

## Notes

All data is available from the corresponding authors upon reasonable request.

## References

[ref1] Debe M. K. Electrocatalyst Approaches and Challenges for Automotive Fuel Cells. Nature 2012, 486 (7401), 43–51. 10.1038/nature11115. 22678278

[ref2] Stamenkovic V. R.; Mun B. S.; Arenz M.; Mayrhofer K. J. J.; Lucas C. A.; Wang G.; Ross P. N.; Markovic N. M. Trends in Electrocatalysis on Extended and Nanoscale Pt-Bimetallic Alloy Surfaces. Nat. Mater. 2007, 6 (3), 241–247. 10.1038/nmat1840. 17310139

[ref3] Strasser P.; Koh S.; Anniyev T.; Greeley J.; More K.; Yu C.; Liu Z.; Kaya S.; Nordlund D.; Ogasawara H.; et al. Lattice-Strain Control of the Activity in Dealloyed Core–Shell Fuel Cell Catalysts. Nat. Chem. 2010, 2 (6), 454–460. 10.1038/nchem.623. 20489713

[ref4] Gasteiger H. A.; Kocha S. S.; Sompalli B.; Wagner F. T. Activity Benchmarks and Requirements for Pt, Pt-Alloy, and Non-Pt Oxygen Reduction Catalysts for PEMFCs. Appl. Catal., B 2005, 56 (1), 9–35. 10.1016/j.apcatb.2004.06.021.

[ref5] Chen C.; Kang Y.; Huo Z.; Zhu Z.; Huang W.; Xin H. L.; Snyder J. D.; Li D.; Herron J. A.; Mavrikakis M.; et al. Highly Crystalline Multimetallic Nanoframes with Three-Dimensional Electrocatalytic Surfaces. Science 2014, 343 (6177), 1339–1343. 10.1126/science.1249061. 24578531

[ref6] Nørskov J. K.; Bligaard T.; Rossmeisl J.; Christensen C. H. Towards the Computational Design of Solid Catalysts. Nat. Chem. 2009, 1 (1), 37–46. 10.1038/nchem.121. 21378799

[ref7] Jia Q.; Zhao Z.; Cao L.; Li J.; Ghoshal S.; Davies V.; Stavitski E.; Attenkofer K.; Liu Z.; Li M.; et al. Roles of Mo Surface Dopants in Enhancing the ORR Performance of Octahedral PtNi Nanoparticles. Nano Lett. 2018, 18 (2), 798–804. 10.1021/acs.nanolett.7b04007. 29272136

[ref8] Li M.; Zhao Z.; Cheng T.; Fortunelli A.; Chen C.-Y.; Yu R.; Zhang Q.; Gu L.; Merinov B. V.; Lin Z.; et al. Ultrafine Jagged Platinum Nanowires Enable Ultrahigh Mass Activity for the Oxygen Reduction Reaction. Science 2016, 354 (6318), 1414–1419. 10.1126/science.aaf9050. 27856847

[ref9] Kühl S.; Gocyla M.; Heyen H.; Selve S.; Heggen M.; Dunin-Borkowski R. E.; Strasser P. Concave Curvature Facets Benefit Oxygen Electroreduction Catalysis on Octahedral Shaped PtNi Nanocatalysts. J. Mater. Chem. A 2019, 7 (3), 1149–1159. 10.1039/C8TA11298C.

[ref10] McCue I.; Benn E.; Gaskey B.; Erlebacher J. Dealloying and Dealloyed Materials. Annu. Rev. Mater. Res. 2016, 46 (1), 263–286. 10.1146/annurev-matsci-070115-031739.

[ref11] Choi S.-I.; Xie S.; Shao M.; Odell J. H.; Lu N.; Peng H.-C.; Protsailo L.; Guerrero S.; Park J.; Xia X.; et al. Synthesis and Characterization of 9 nm Pt–Ni Octahedra with a Record High Activity of 3.3 A/mgPt for the Oxygen Reduction Reaction. Nano Lett. 2013, 13 (7), 3420–3425. 10.1021/nl401881z. 23786155

[ref12] Cui C.; Gan L.; Li H.-H.; Yu S.-H.; Heggen M.; Strasser P. Octahedral PtNi Nanoparticle Catalysts: Exceptional Oxygen Reduction Activity by Tuning the Alloy Particle Surface Composition. Nano Lett. 2012, 12 (11), 5885–5889. 10.1021/nl3032795. 23062102

[ref13] Stamenkovic V. R.; Fowler B.; Mun B. S.; Wang G.; Ross P. N.; Lucas C. A.; Markovic N. M. Improved Oxygen Reduction Activity on Pt_3_Ni(111) via Increased Surface Site Availability. Science 2007, 315 (5811), 493–497. 10.1126/science.1135941. 17218494

[ref14] Wu J.; Gross A.; Yang H. Shape and Composition-Controlled Platinum Alloy Nanocrystals Using Carbon Monoxide as Reducing Agent. Nano Lett. 2011, 11 (2), 798–802. 10.1021/nl104094p. 21204581

[ref15] Yin Y.; Alivisatos A. P. Colloidal Nanocrystal Synthesis and the Organic–Inorganic Interface. Nature 2005, 437 (7059), 664–670. 10.1038/nature04165. 16193041

[ref16] Talapin D. V.; Lee J.-S.; Kovalenko M. V.; Shevchenko E. V. Prospects of Colloidal Nanocrystals for Electronic and Optoelectronic Applications. Chem. Rev. 2010, 110 (1), 389–458. 10.1021/cr900137k. 19958036

[ref17] Murray C. B.; Kagan C. R.; Bawendi M. G. Synthesis and Characterization of Monodisperse Nanocrystals and Close-Packed Nanocrystal Assemblies. Annu. Rev. Mater. Sci. 2000, 30 (1), 545–610. 10.1146/annurev.matsci.30.1.545.

[ref19] Stamenkovic V.; Mun B. S.; Mayrhofer K. J. J.; Ross P. N.; Markovic N. M.; Rossmeisl J.; Greeley J.; Nørskov J. K. Changing the Activity of Electrocatalysts for Oxygen Reduction by Tuning the Surface Electronic Structure. Angew. Chem., Int. Ed. 2006, 45 (18), 2897–2901. 10.1002/anie.200504386. 16596688

[ref20] Wang R.; Wang H.; Luo F.; Liao S. Core–Shell-Structured Low-Platinum Electrocatalysts for Fuel Cell Applications. Electrochem. Energy Rev. 2018, 1 (3), 324–387. 10.1007/s41918-018-0013-0.

[ref21] Lim S. I.; Ojea-Jiménez I.; Varon M.; Casals E.; Arbiol J.; Puntes V. Synthesis of Platinum Cubes, Polypods, Cuboctahedrons, and Raspberries Assisted by Cobalt Nanocrystals. Nano Lett. 2010, 10 (3), 964–973. 10.1021/nl100032c. 20143792

[ref22] Wang Y.-C.; Slater T. J. A.; Leteba G. M.; Roseman A. M.; Race C. P.; Young N. P.; Kirkland A. I.; Lang C. I.; Haigh S. J. Imaging Three-Dimensional Elemental Inhomogeneity in Pt–Ni Nanoparticles Using Spectroscopic Single Particle Reconstruction. Nano Lett. 2019, 19 (2), 732–738. 10.1021/acs.nanolett.8b03768. 30681878 PMC6378652

[ref23] Chang Q.; Xu Y.; Duan Z.; Xiao F.; Fu F.; Hong Y.; Kim J.; Choi S.-I.; Su D.; Shao M. Structural Evolution of Sub-10 nm Octahedral Platinum–Nickel Bimetallic Nanocrystals. Nano Lett. 2017, 17 (6), 3926–3931. 10.1021/acs.nanolett.7b01510. 28493711

[ref25] Shui J. I.; Chen C.; Li J. C. M. Evolution of Nanoporous Pt-Fe Alloy Nanowires by Dealloying and Their Catalytic Property for Oxygen Reduction Reaction. Adv. Funct. Mater. 2011, 21 (17), 3357–3362. 10.1002/adfm.201100723.

[ref26] Maillard F.; Schreier S.; Hanzlik M.; Savinova E. R.; Weinkauf S.; Stimming U. Influence of Particle Agglomeration on the Catalytic Activity of Carbon-Supported Pt Nanoparticles in CO Monolayer Oxidation. Phys. Chem. Chem. Phys. 2005, 7 (2), 385–393. 10.1039/B411377B.

[ref27] Cui C.; Gan L.; Heggen M.; Rudi S.; Strasser P. Compositional Segregation in Shaped Pt Alloy Nanoparticles and Their Structural Behaviour during Electrocatalysis. Nat. Mater. 2013, 12, 765 10.1038/nmat3668. 23770725

[ref28] Snyder J.; Livi K.; Erlebacher J. Oxygen Reduction Reaction Performance of [MTBD][Beti]-Encapsulated Nanoporous NiPt Alloy Nanoparticles. Adv. Funct. Mater. 2013, 23 (44), 5494–5501. 10.1002/adfm.201301144.

[ref29] Shao M.; Chang Q.; Dodelet J.-P.; Chenitz R. Recent Advances in Electrocatalysts for Oxygen Reduction Reaction. Chem. Rev. 2016, 116 (6), 3594–3657. 10.1021/acs.chemrev.5b00462. 26886420

[ref30] Ehteshami S. M. M.; Chan S. H. A Review of Electrocatalysts with Enhanced CO Tolerance and Stability for Polymer Electrolyte Membarane Fuel Cells. Electrochim. Acta 2013, 93, 334–345. 10.1016/j.electacta.2013.01.086.

[ref31] Chung D. Y.; Yoo J. M.; Sung Y. Highly Durable and Active Pt-Based Nanoscale Design for Fuel-Cell Oxygen-Reduction Electrocatalysts. Adv. Mater. 2018, 30 (42), 1704123 10.1002/adma.201704123. 29359829

[ref32] Huang X.; Zhao Z.; Cao L.; Chen Y.; Zhu E.; Lin Z.; Li M.; Yan A.; Zettl A.; Wang Y. M.; et al. High-Performance Transition Metal-Doped Pt_3_Ni Octahedra for Oxygen Reduction Reaction. Science 2015, 348 (6240), 1230–1234. 10.1126/science.aaa8765. 26068847

[ref33] Lim J.; Shin H.; Kim M.; Lee H.; Lee K.-S.; Kwon Y.; Song D.; Oh S.; Kim H.; Cho E. Correction to Ga–Doped Pt–Ni Octahedral Nanoparticles as a Highly Active and Durable Electrocatalyst for Oxygen Reduction Reaction. Nano Lett. 2018, 18 (8), 5343–5343. 10.1021/acs.nanolett.8b02845. 30014701

[ref35] Zhang S.; Yuan X.-Z.; Hin J. N. C.; Wang H.; Friedrich K. A.; Schulze M. A Review of Platinum-Based Catalyst Layer Degradation in Proton Exchange Membrane Fuel Cells. J. Power Sources 2009, 194 (2), 588–600. 10.1016/j.jpowsour.2009.06.073.

[ref39] Li X.; Chen Q.; McCue I.; Snyder J.; Crozier P.; Erlebacher J.; Sieradzki K. Dealloying of Noble-Metal Alloy Nanoparticles. Nano Lett. 2014, 14 (5), 2569–2577. 10.1021/nl500377g. 24689459

[ref40] Gan L.; Heggen M.; O’Malley R.; Theobald B.; Strasser P. Understanding and Controlling Nanoporosity Formation for Improving the Stability of Bimetallic Fuel Cell Catalysts. Nano Lett. 2013, 13 (3), 1131–1138. 10.1021/nl304488q. 23360425

