# Supplementary material for: Oleylamine Aging of PtNi Nanoparticles Giving Enhanced Functionality for the Oxygen Reduction Reaction
Source: Nano Lett. 2021 Apr 26;21(9):3989–96. doi: 10.1021/acs.nanolett.1c00706 (PMC8289299; doi:10.1021/acs.nanolett.1c00706)
Supplement: Supplementary file 1 — nl1c00706_si_001.pdf [file nl1c00706_si_001.pdf]

Supporting Information for

"Oleylamine Aging of PtNi Nanoparticles Giving Enhanced Functionality for the Oxygen Reduction Reaction"

Gerard M Leteba[1,8], Yi-Chi Wang[2,9,10], Thomas J A Slater[2,3], Rongsheng Cai[2], Conor Byrne[4,11], Christopher P Race[2], David R G Mitchell[5], Pieter B J Levecque[1], Neil P Young[6], Stuart M Holmes[7], Alex Walton[4,11], Angus I Kirkland[3,6], Sarah J Haigh[2*], Candace I Lang[8*]

1. Catalysis Institute, Department of Chemical Engineering, University of Cape Town, Corner of Madiba Circle and South Lane, Rondebosch 7701, South Africa.
2. Department of Materials, University of Manchester, Manchester, M13 9PL, UK.
3. Electron Physical Sciences Imaging Centre, Diamond Light Source Ltd., Oxfordshire OX11 0DE, UK
4. Department of Chemistry, University of Manchester, Manchester, M13 9PL, UK
5. Electron Microscopy Centre, Innovation Campus, University of Wollongong, Wollongong NSW 2517, Australia
6. Department of Materials, University of Oxford, Parks Road, Oxford, OX1 3PH, U.K.
7. Department of Chemical Engineering and Analytical Science, University of Manchester, M13 9PL, UK.
8. School of Engineering, Macquarie University, NSW 2109 Australia
9. Beijing Institute of Nanoenergy and Nanosystems, Chinese Academy of Sciences, Beijing, 101400, China
10. School of Nanoscience and Technology, University of Chinese Academy of Sciences, Beijing, 100049, China
11. Photon Science Institute, University of Manchester, Manchester M13 9PL, United Kingdom

*sarah.haigh@manchester.ac.uk
*candace.lang@mq.edu.au

Experimental Methods:

Synthesis of nanoparticles.

In a standard co-reduction procedure: 0.03 g nickel (II) acetate tetrahydrate ($Ni(Ac)_2{\cdot}4H_2O$) and 0.09 g chloroplatinic acid solution (H_2PtCl_6, 8 wt. % in water) (precursor salts), 15 ml oleylamine (OAm), 4.4 g octadecylamine (ODA) and 15 ml oleic acid (OLEA) (hydrophobic surfactants) were dissolved in 25 ml 1-octadecene (1-OD) (a high boiling point solvent) by sonication for 20 minutes; then heated at 150 °C under vigorous magnetic stirring until a pale yellow solution was observed. After the addition of 0.05 g tetrabutylammonium borohydride (TBAB) as the reductant, the reaction temperature was raised to 240 °C and maintained for 30–40 minutes in air. (The effect of the surfactant OLEA was investigated by repeating this protocol, replacing OLEA with TOA). The nanoparticles were aged in OAm for 3 weeks , followed by the addition of excess chloroform, purification by the addition of ethanol and finally, re-dispersing the particles in chloroform by vigorous sonication. The separation-precipitation washing process was performed three times to eliminate any unbound surfactants on the surfaces of the nanoparticles. Thereafter the black product was dried and finally re-suspended in chloroform. To further investigate the influence of reaction conditions the 1-OD solvent was substituted by an equal volume of benzyl ether (BE). This substitution also had no observable effect on the morphology of the nanoparticles produced (Figure S2).

We observe that the use of OAm in the initial co-reduction procedure results in excellent redispersion of the synthesized nanoparticles (required for detailed TEM analysis and for the homogeneous deposition on support materials) and also reverses the storage-instability of agglomerated nanoparticles. We further observe that this peptization phenomenon is only apparent in nanoparticles which have been solution-grown using hydrophobic surface coordinating surfactants. Unlike OAm, the use of organic nanoparticle stabilizers TOA and OLEA as the re-dispersants did not deflocculate the nanoparticle aggregates.

Deposition of nanomaterials onto carbon support.

The as-prepared nanostructures were dispersed onto a carbon support (Cabot, Vulcan XC-72R) via a colloidal-deposition strategy, by mixing these nanomaterials and chloroform, followed by sonication for 15–20 min. The resulting homogeneous reaction dispersion was left in a fume hood overnight to evaporate the chloroform. The resultant carbon-supported materials were further washed with acetone 3–4 times and dried in an oven at 60 °C. The metal loading (wt. % metal) for each sample was verified using thermogravimetric analysis (Mettler Toledo TGA/sDTA851e).

Nanostructure characterization techniques.

The black powders of the as-synthesised nanostructures were deposited onto a zero-background silicon (Si) wafer support and characterized by powder XRD on an X'Pert Pro multipurpose diffractometer

(MPD), using Cu K_α radiation ($\lambda = 0.154056$ nm). The diffraction patterns were recorded at a scan rate of 0.106°/s and with a step size of 0.0334°. Specimens for scanning transmission electron microscopy (STEM) investigations were prepared by drop-casting a colloidal solution onto 3-mm carbon-supported films on copper grids. These were air dried under ambient conditions. The uniformity in size and shape of the nanoparticles favours their self-arrangement/assembly into superlattice rafts when dispersed onto a thin carbon film suitable for transmission electron microscope (TEM) imaging. STEM-HAADF and EDS data were collected using a Thermo Fisher Titan G2 80-200 S/TEM operated at 200 kV, which was equipped with an X-FEG high brightness source, STEM probe aberration correction and a ChemiSTEM™ Super-X EDS detector consisting of four silicon drift detectors (SDDs) with a total collection solid angle of approximately 0.7 sr. The average chemical composition of the nanoparticles was measured by summing the STEM-EDXS spectra obtained for individual particles and quantifying using a Cliff-Lorimer analysis (Figure S**6**). A convergence angle of 21 mrad and an acceptance inner angle of 55 mrad were used for HAADF STEM image acquisition. Tilt-series tomography HAADF images were collected using a Fischione 2020 single tilt tomography holder and FEI Inspect3D software. The total tilt range was ±70° with a pixel size of 0.07 nm and a pixel dwell time of 10 µs. Incremental steps of 2° at ±50° and 1° for the rest of the tilt range were used. Nanoparticle size refers to the particle's diameter. To measure this from an HRTEM image of an individual particle two orthogonal lines were drawn across the particle, the first being aligned with the particle's largest dimension. The diameter was then determined as the average of the two line lengths.

The 3D reconstruction shown in Figure 2 was performed using a single particle reconstruction method, averaging approximately 400 PtNi-OLEA-Aged nanoparticles. As these nanoparticles are similar in shape and randomly oriented on the support, several STEM-HAADF images of different areas can collect data for about 1000 individual nanoparticles at various orientations. Nanoparticle orientations were solved by cross-correlating the experimental images with the reprojections from a conventional tilt series tomogram. Fourier-space back projection was used for 3D reconstruction after the orientation of each HAADF image was assigned. Detailed methods for this single particle reconstruction technique of these inorganic nanoparticles are described elsewhere.[1] Simulation of HRTEM images was performed using QSTEM[2] with the atomic model as an input with parameters of 200 kV accelarating voltage, 61.3 nm defocus, 0.5 mrad convergence angle, 3 nm focal spread, 0 nm astigmatism, 1 mm 3rd order speherical aberration and amorphous noise. To measure the local interatomic spacings from HAADF STEM data, each atom column position was identified using 2D Gaussian fitting in the python package atomap.[3] For each atom column, the distances to its surrounding atoms were calculated and averaged. This distance was then used to colour the atomic columns.

Specimens for X-ray photoelectron spectroscopy (XPS) were prepared by dropcasting suspensions (dispersed in chloroform) onto silicon wafers. High Resolution XPS Spectra were taken using a SPECS NAP-XPS instrument (operating under UHV conditions, base pressure of 1×10^{-9} mbar). XPS

measurements were taken using a microfocussed, monochromated Al K_α X – Ray source (1487eV) and a SPECS Phoibos NAP 150 hemispherical analyser. Scans were taken at normal emission and a pass energy of 30 eV for detailed scans and 60 eV for survey scans. The samples were charge referenced to adventitious carbon at 284.8 eV. As shown in Figure S8 in the as-prepared condition, PtNi nanoparticles exhibit a double peak in the Pt 4f region, corresponding to metallic Pt, which is also evident in aged samples. A small shift in position of the major Pt peak is observed after aging, reflecting a reduction in Ni content,[4] as also observed via TEM and PXRD after aging. Only a small peak corresponding to metallic Ni was detected (at around 852 eV) in as-prepared samples; this weak Ni peak is no longer evident in the C supported particles after aging. XPS spectra in Figure S8 also showed that the metallic Ni peak, visible in as-prepared nanoparticles, was absent in the C-supported nanoparticles after aging and after durability testing. The Pt peak remained well defined on the supported materials even after durability testing. A small Pt peak shift (from 71.0 eV to 71.5 eV) was observed after durability testing, which may suggest a decrease in Pt at the particle surface[5] although this was not detectable by STEM-EDXS.

Electrochemical Measurements.

All experiments were performed in a standard three electrode setup at room temperature in a 0.1 M perchloric acid ($HClO_4$, 70%) solution using either argon (Ar) 99.999% (Afrox), oxygen (O_2) 99.998% (Afrox) and carbon dioxide (CO) 99% (Afrox) as specified. A platinum (Pt) coil was used as the counter electrode. A mercury/mercurous sulphate reference electrode was used as a reference and all potential values were reported relative to the standard hydrogen electrode (SHE). The readout currents were not corrected for the ohmic iR losses. A Biologic SP300 potentiostat was coupled to a RDE710 Rotator (Gamry instrument). Catalyst inks were prepared by mixing 10 mg of the catalyst with 2 ml of Milli-Q water (Millipore, 18 MΩ.cm @ 25 °C), 0.45 ml isopropanol and 25 µl Nafion® perfluorinated resin solution (5 wt.% in a mixture of lower aliphatic alcohols and water (45% water)). The resultant mixture was sonicated for 15–20 min. 10 µl of the ink was then pipetted onto a glassy carbon (GC) electrode (Pine Research Instrumentation, 5 mm disk OD) and dried under ambient conditions for 30–60 minutes to evaporate the solvents. The remaining thin black uniform film of Nafion-catalyst-Vulcan on the GC served as the working electrode (WE). Before use the GC electrode was polished to a mirror finish on a Microcloth polishing pad (Buehler) using 1 µm and 0.05 µm alumina paste (Buehler). After rinsing, the WE was ultrasonicated in Milli-Q water (Millipore, 18 MΩ.cm @ 25 °C) for 10 min and left to dry.

(a) Cyclic voltammetry.

In an Ar purged electrolyte, the potential of the WE was cycled between 0.05 V and 1.00 V vs. SHE at a scan rate of 100 mV/s for 30 cycles to electrochemically clean the catalyst surface. The sweep rate was then reduced to 50 mV/s and the third cycle at that scan rate was used for analysis. The electrochemically active surface area (ECSA) was calculated by integrating the area under the curve for

the hydrogen underpotential deposition region (H_{upd}) assuming a monolayer hydrogen charge of 210 $\mu C/cm^2_{Pt}$.[6-7]

(b) Carbon dioxide (CO) stripping voltammetry.

The CV curves show the hydrogen adsorption/desorption (~0.05–0.35 V, *vs* standard hydrogen electrode, (SHE)) and oxide formation/reduction (~ 0.70–1.00 V, *vs* SHE) curves. CO gas was bubbled into the electrolyte solution while holding the potential of the working electrode at 0.1 V vs. SHE. The electrolyte was then purged with Ar to remove the dissolved CO gas while still holding the potential of the WE at 0.1 V vs. SHE. The potential of the WE was then cycled to 1.00 V vs. SHE at 20 mV/s, followed by a CV cycle as described above at 20 mV/s. The peak area could then be determined using the baseline CV and a normalisation factor of 420 $\mu g/cm^2_{Pt}$[8] was used to calculate the ECSA.

(c) Linear Sweep Voltammetry.

The potential of the WE was swept from 1.10 V to 0.20 V vs. SHE and back at 10 mV/s. ORR polarization curves were recorded at rotation speeds of 400, 900, 1600 and 2500 rpm. The ORR curves obtained in O_2 saturated electrolyte were corrected for the capacitive current associated with Pt_xM_y/C catalysts, by subtracting a CV curve measured in an argon-saturated electrolyte. All the polarization curves reported in this work were acquired using a rotation speed of 1600 rpm and the current densities were also normalized with reference to the calculated ECSA to evaluate the specific activities.

The current density (*i*) for the ORR electrocatalytic activity is calculated according to Koutecky-Levich equation:[9-10]

$$\frac{1}{i} = \frac{1}{i_k} + \frac{1}{i_d}$$

where i is the overall disk current density, i_k is the true kinetic current density (A) and is determined by the mass transport properties of the RDE, i_d is the diffusion limited current density. i_d can be expressed according to Levich equation as follows:

$$i_d = 0.201 n_e F A D_{O_2}{}^{2/3} V^{-1/6} C_{O_2}{}^{\infty} \omega^{1/2}$$

where n_e is the total number of electrons transferred ($4e^-$), F is the Faraday's constant (96485 C/mol), A is the surface area of the electrode (0.196 cm^2), D_{O_2} is the diffusion coefficient of oxygen (1.93×10^{-5} cm^2/s), C_{O_2} is the concentration of dissolved oxygen (1.26×10^{-6} mol/cm^3), V is the kinematic viscosity of the electrolyte solution (1.01×10^{-2} cm^2/s) at 20 °C and ω is the angular frequency of rotation, $\omega = \frac{2\pi f}{60}$, f is the RDE rotation rate in rpm: for the measurements conducted in 0.1 M $HClO_4$ at 20 °C and 1 atm O_2. The diffusion coefficient of oxygen, the kinematic viscosity of the electrolyte solution and the concentration of dissolved oxygen are classified as non-electrochemical kinetic characteristics required for RDE data analysis. These kinetic parameters are influenced by temperature and the electrolyte solution during the electrochemical measurements.[11]

In order to determine the mass-transport free kinetic current (i_k), the ORR measurements are conducted at the rotation speed of 1600 rpm. This rotation rate is used as the benchmark to compare the functionality of electrocatalysts and the ORR polarization limiting current ranges between 5.8×10^{-3} – 6×10^{-3} mA cm^{-2}.[9, 12] These limiting current values yield n = 4 for the Levich and Levich-Koutecky plots. Furthermore, background current measurements are performed in deaerated electrolyte solution to account for capacitive current interferences. The difference between the experimentally measured current and the background current yields mass-transport corrected current. This current is used to evaluate mass-and area-specific activities of catalysts. Since at the limiting current the reaction kinetics occur very fast, the Koutecky-Levich equation can be re-arranged as follows:[10, 12-13]

$$I_k\,(A) = \frac{I_{lim}\,(A)\ x\ I(A)}{(I_{lim} - I)\ (A)}$$

where $I_{lim} = i_d$ is the measured diffusion limited current density and I_k is the kinetic current (A). The I and I_{lim} are the values calculated from the anodic ORR polarization curve at E = 0.9 V and E = 0.4 V versus SHE, respectively.[13]

The Pt mass-specific (I_m) and area-specific (I_s) activities are quantified at E = 0.9 V versus SHE specifically because the contributions from mass-transport losses cannot be totally disregarded at the higher current densities detected below E = 0.9 V.[11-14] Therefore, the Pt mass-specific activity is calculated from the I_k and normalization to the Pt-loading of the GC disk electrode:[11-13]

$$I_{m(0.90V)}(A\ \text{mg}_{\text{Pt}}^{-1}) = \frac{I_k\,(A)}{L_{\text{Pt}}(\text{mg}_{\text{Pt}}\ \text{cm}^{-2})(\text{Ag cm}^2)}$$

L_{Pt} is the working electrode catalyst loading ($mg_{catalyst}$ cm^{-2}) and Ag (cm^2) is the geometric surface area of the glassy carbon electrode. The area-specific activity is determined from the I_k and normalization with the Pt electrochemical surface area (ECSA):[12-13, 15]

$$I_{s(0.90V)}(mA/cm^2) = \frac{I_k\,(A)}{ECSA}$$

$$= \frac{I_k\,(A)}{(\text{Q}_{\text{H-desorption (or adsorption)}}(\text{C})/210\ \mu\text{C cm}^{-2})Ag(\text{cm}^2)}$$

(d) Fourier transform infrared (FT-IR) spectroscopy to analyse the influence of surfactants

In order to investigate the possible influence of surfactants on ECSA, we conducted Fourier transform infrared (FT-IR) measurements on all samples, as shown in Figure S13. The FT-IR spectra are in good agreement with previous reports.[16-17] The spectra of as-synthesized PtNi and aged PtNi nanoparticles shown in Figure S13 are similar to that of OAm, but not to OLEA, ODA or TOA. These FT-IR spectral investigations thus suggest that the organic layers passivating the surfaces of all nanoparticles (after repeated destabilization/purification processes) are only OAm. We would therefore expect only OAm to have a possible influence on the ECSA measurements. However, the higher ECSA values and

significant positive shifts in the ORR polarization curves, in addition to the well-defined diffusion limited current density plateaux (reaching the theoretical value of ~6 mA/cm^2) suggest minimal interference of OAm during the catalytic performance of these PtNi systems. We therefore conclude that the influence of surfactants on catalytic activity is not significant. We also observe shifts in the diffusion limited current density regime from ~6.0 to ~5.8 mA/cm^2 post 5000 electrochemical cycles, suggesting restricted mass transport (O_2) diffusion and this may be induced by these electrocatalysts microstructural deformations or blockage/deactivation of Pt active sites due to surface contamination (Figure S10).

(e) Durability testing

Durability tests were conducted by subjecting the working electrode for the Aged-electrocatalysts to potential cycling between 0.05 V and 1.00 V at a scan rate of 100 mV/s for 5000 cycles in an Ar-purged 0.1 M $HClO_4$ electrolyte solution. CV measurements after 5000 electrochemical cycles of long-term in-service tests show apparent decrease in both the hydrogen desorption/adsorption and oxide formation/reduction peaks. The evolution of two prominent hydrogen adsorption (H_{ads}) current peaks, ascribed to {100} and {110} facets, corresponds to that of pure polycrystalline Pt[18] (Figure S10). Thus, the $ECSAs_{Hupd}$ of our nanoalloys decreased, relative to their initial value, by 40% for PtNi-OLEA-Aged/C and 36% for PtNi-TOA-Aged/C (Figure S11 and Table S2). In addition, recorded CO stripping curves display significant drop in current peaks ($ECSA_{CO}$ loss of 40% for PtNi-OLEA-Aged/C and 33% for PtNi-TOA-Aged/C) and positive potential shift (from lower to higher) (Figure S10 and Table S2).

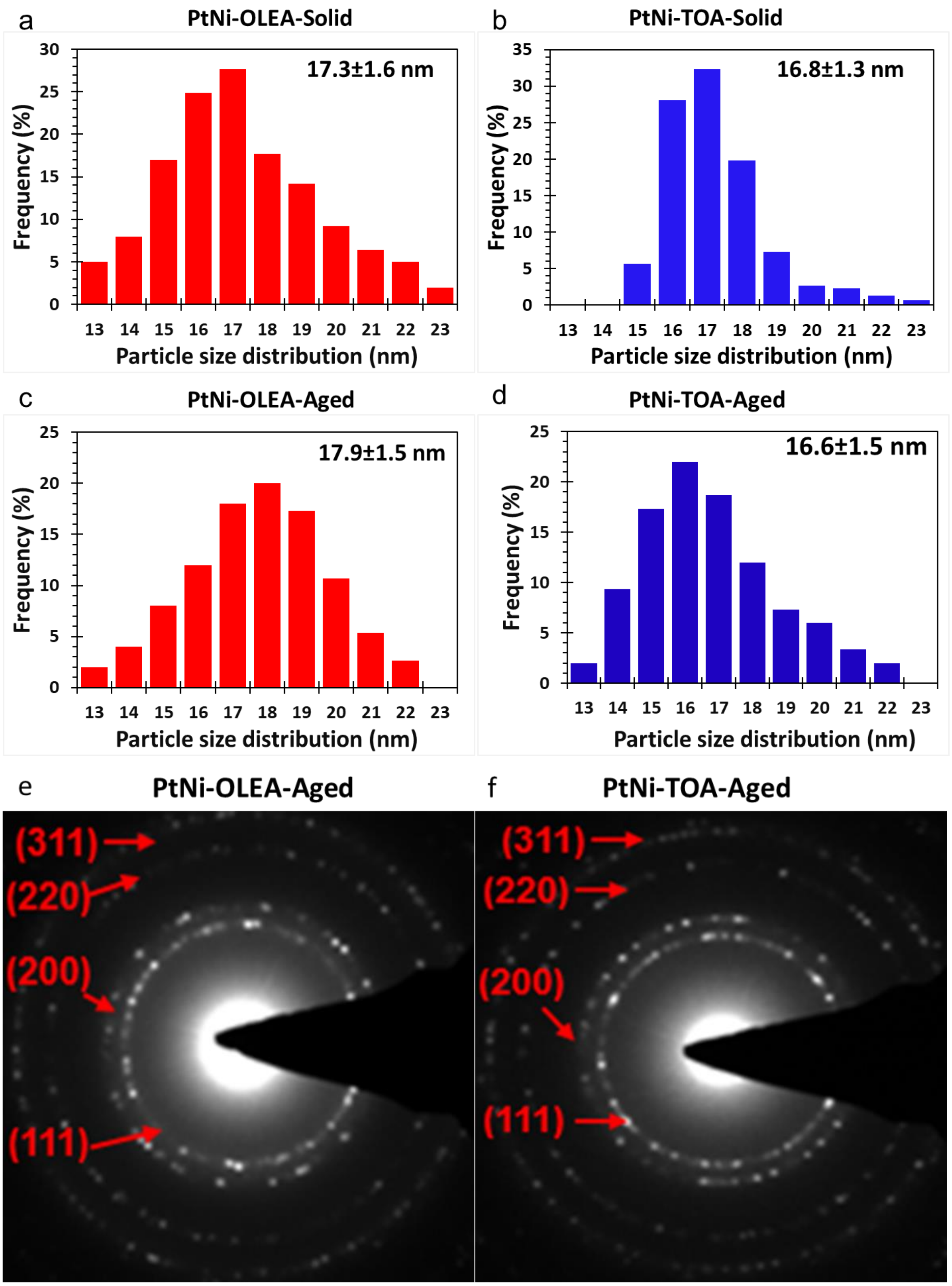

Figure S1. Nanostructures synthesized using high boiling point solvent 1-octadecene. (**a-d**) size distribution histograms of PtNi-OLEA-Solid, PtNi-TOA-Solid, PtNi-OLEA-Aged and PtNi-TOA-Aged nanoparticles, respectively. (**e,f**) Selected area electron diffraction patterns for both PtNi-OLEA-Aged and PtNi-TOA-Aged nanoparticles indicate the same dominant crystallographic features as those found in PXRD.

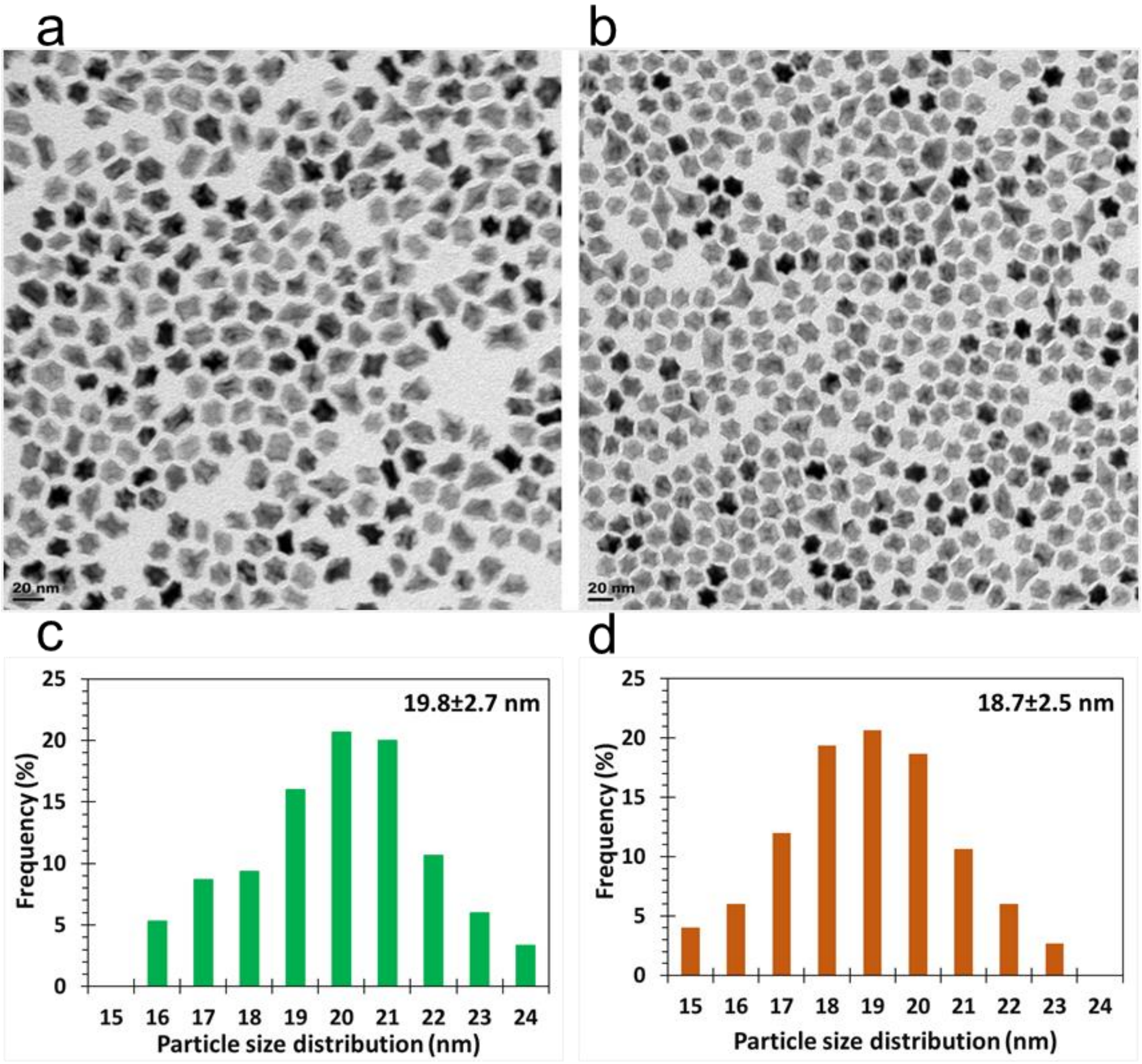

Figure S2. Nanostructures synthesized using a high-boiling point solvent benzyl ether, showing excellent dispersity. (**a,b**) Overview TEM images of PtNi-OLEA-Aged and PtNi-TOA-Aged, respectively. (**c,d**) size distribution histograms of PtNi-OLEA-Aged and PtNi-TOA-Aged, respectively.

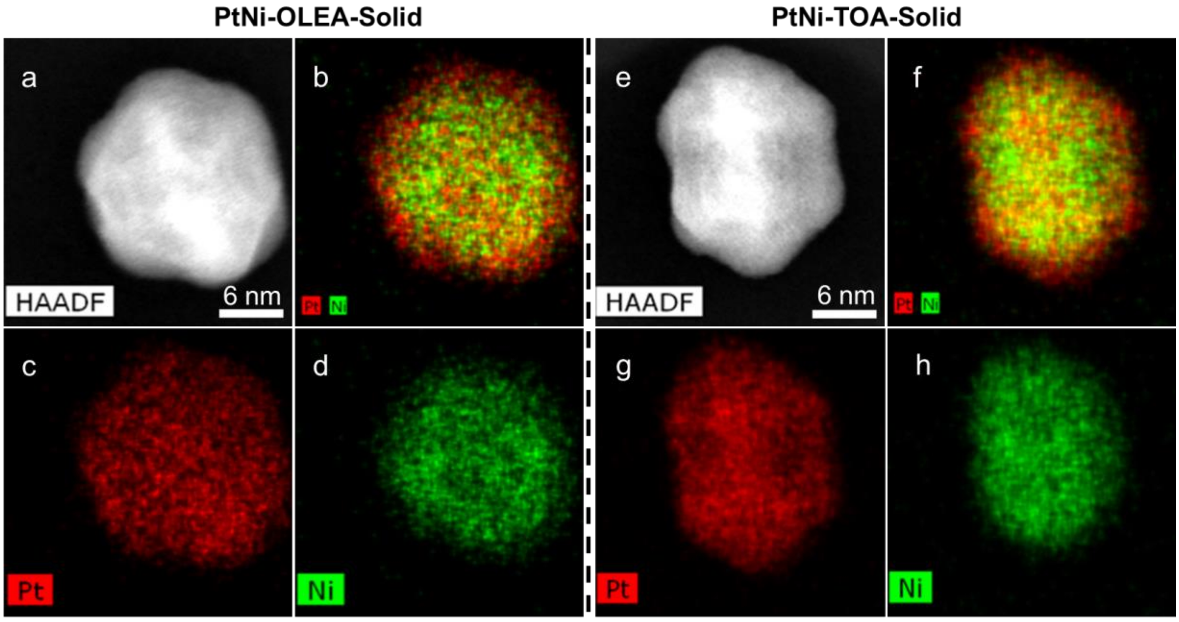

Figure S3. STEM-EDXS analysis of elemental distribution for **(a-d**) PtNi-OLEA-Solid nanoparticles and (**e-h**) PtNi-TOA-Solid nanoparticles. (**a,e**) show HAADF STEM images, (**b,f**) are EDXS composite maps of Pt and Ni, (**c,g**) are EDXS Pt maps and (**d,h**) EDXS Ni maps.

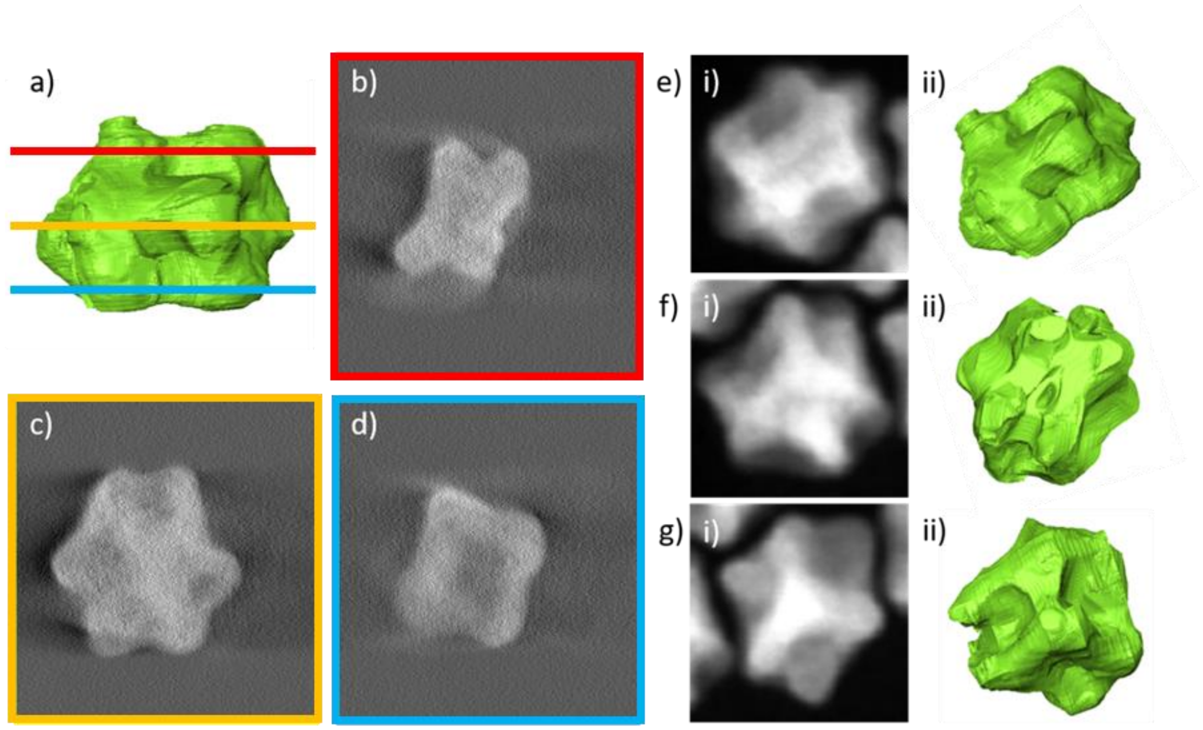

Figure S4. STEM HAADF tomography reconstruction of a PtNi-OLEA-Aged nanoparticle. (**a**) Surface visualisation of the nanoparticle and slices through the (**b**) top, (**c**) middle and (**d**) bottom of the nanoparticle, as indicated by the lines in (**a**). The reconstruction demonstrates a clear rhombic dodecahedron structure. (**e-f**) Comparison of HAADF STEM images (**i**) with surface visualisations (**ii**) of the tomographic reconstruction in a similar orientation.

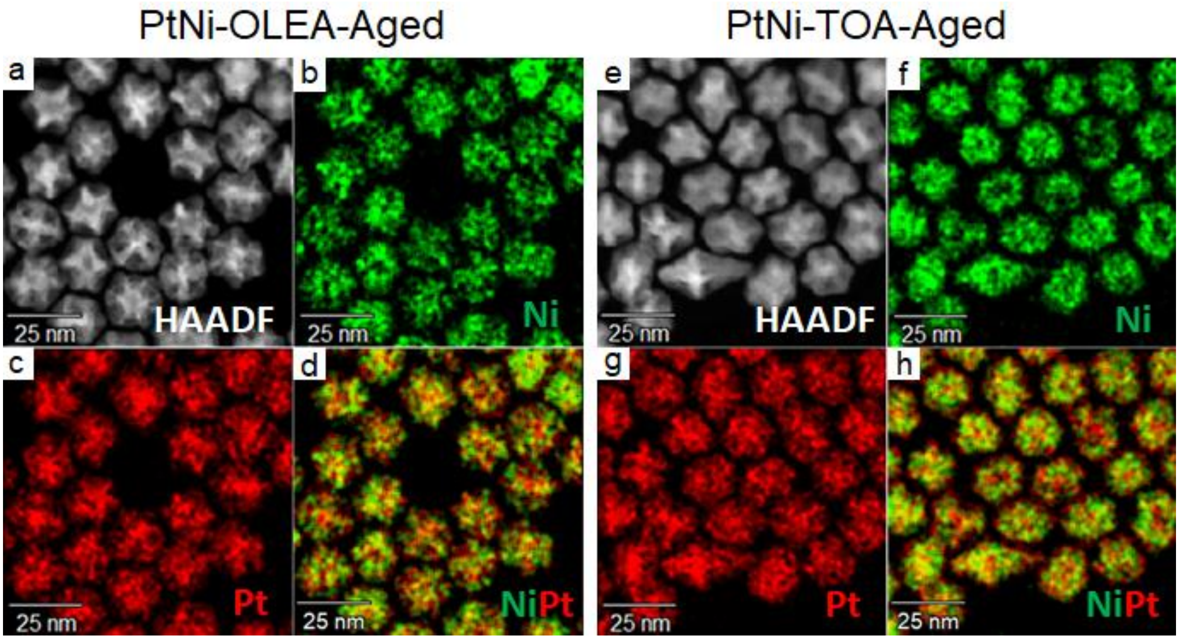

Figure S5. STEM-EDXS analysis of elemental distribution for (**a-d**) PtNi-OLEA-Aged nanoparticles and (**e-h**) PtNi-TOA-Aged nanoparticles. (**a,e**) show HAADF STEM images, (**b,f**) are EDXS Ni maps, (**c,g**) EDXS Pt maps and (**d,h**) EDXS composite maps of Pt and Ni.

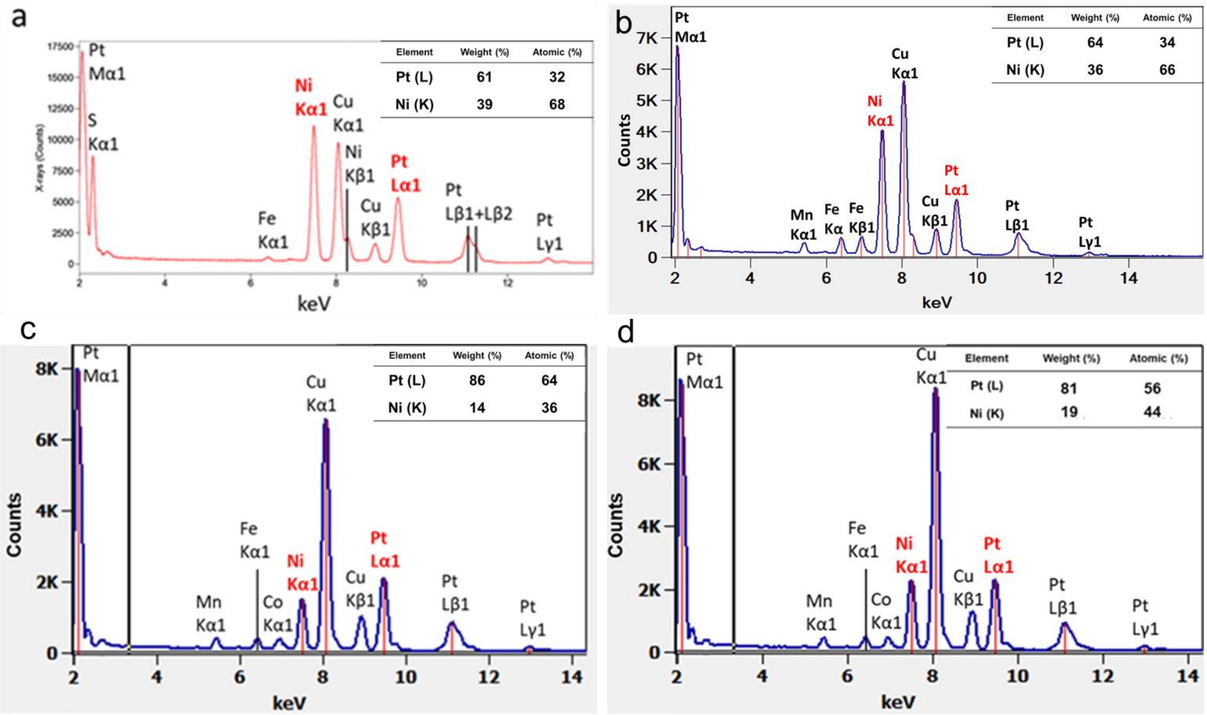

Figure S6. EDX spectra of (**a**) PtNi-OLEA-Solid, (**b**) PtNi-TOA-Solid, (**c**) PtNi-OLEA-Aged and (**d**) PtNi-TOA-Aged nanoparticles. All detected X-ray peaks in the energy range of 2 keV to 14 keV are labelled. Peaks in range of 0-2 keV are not shown due to the large number of overlapping elements present in this energy range. The Cu $K_{\alpha1}$ and Cu $K_{\beta1}$ peaks result from the use of a Cu TEM support grid, Mn $K_{\alpha1}$ from the use of a Mn washer to secure the grid. Peaks from Fe and Co are artefacts due to scattering from the polepiece. The presence of S $K_{\alpha1}$ in the PtNi-OLEA-Solid EDX spectrum is expected to be due to contamination. Major peaks used for quantification of the nanoparticle composition are labelled in red.

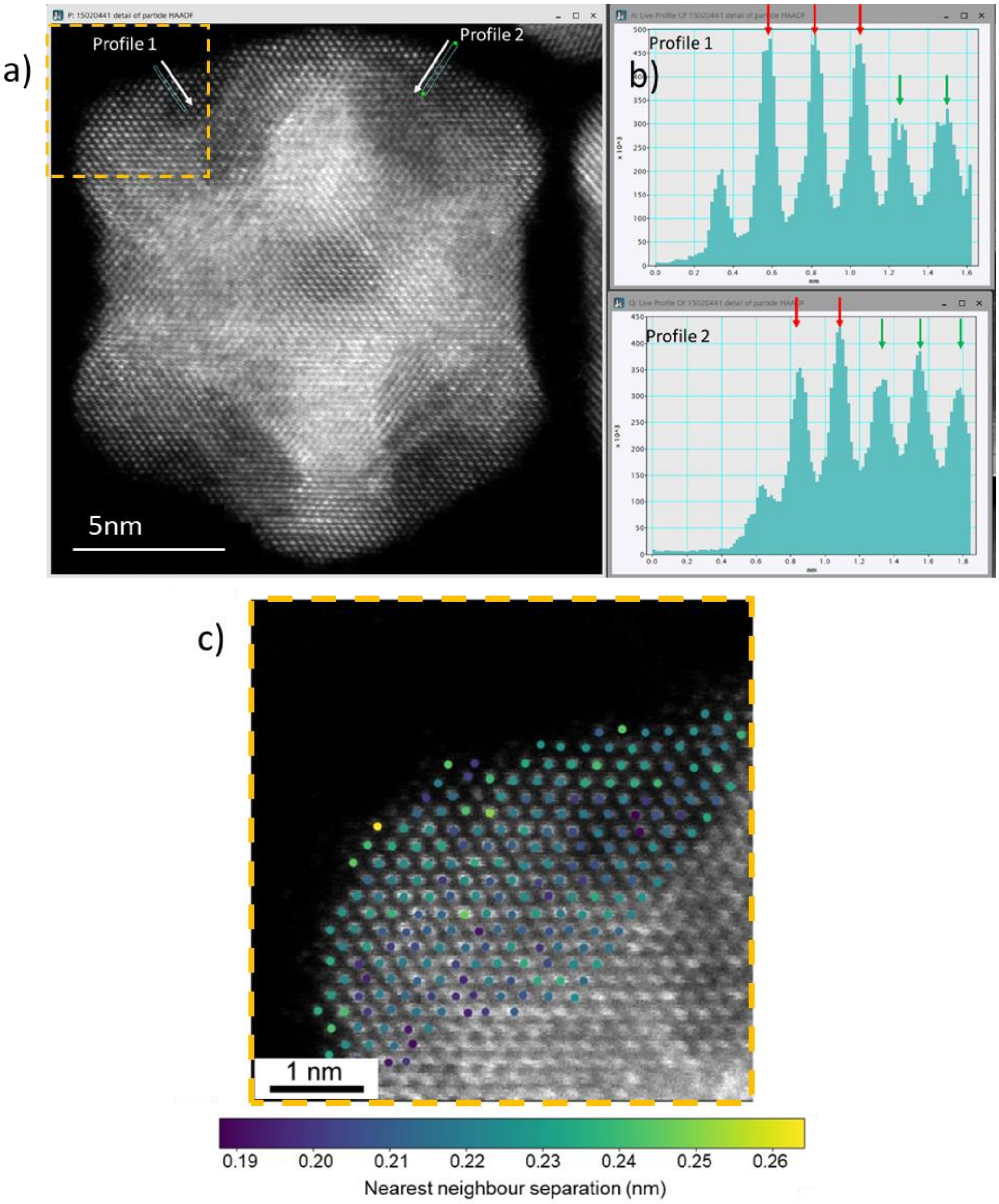

Figure S7. Atom column intensity and position analysis for a PtNi-TOA-Aged nanoparticle. a) HAADF STEM image, b) line profiles from the positions marked as white arrows and cyan rectangles in (a). On the profiles red arrows indicate atomic columns close to the surface of the sample where the HAADF intensity increases unexpectedly. For uniform composition the thickness is expected to decrease towards the edge of the particle, so this suggests these surface layers contain more of the higher atomic number species (Pt). Thus the Pt rich surface layer is estimated as ~3-4 atomic columns; consistent with the Pt enrichment observed in STEM-EDXS mapping. c) Analysis of projected nearest neighbour distance for a region of the particle shown by the dashed orange square in (a). Here we measure an average value of ~0.22 ±0.01 nm, consistent with the expected value for the {111} spacing in PtNi-TOA-Aged particles as measured via PXRD and electron diffraction (~0.220 nm see table S1). Qualitatively slightly larger interlayer spacings appear to be associated with the surface layer (3-4 atomic columns).

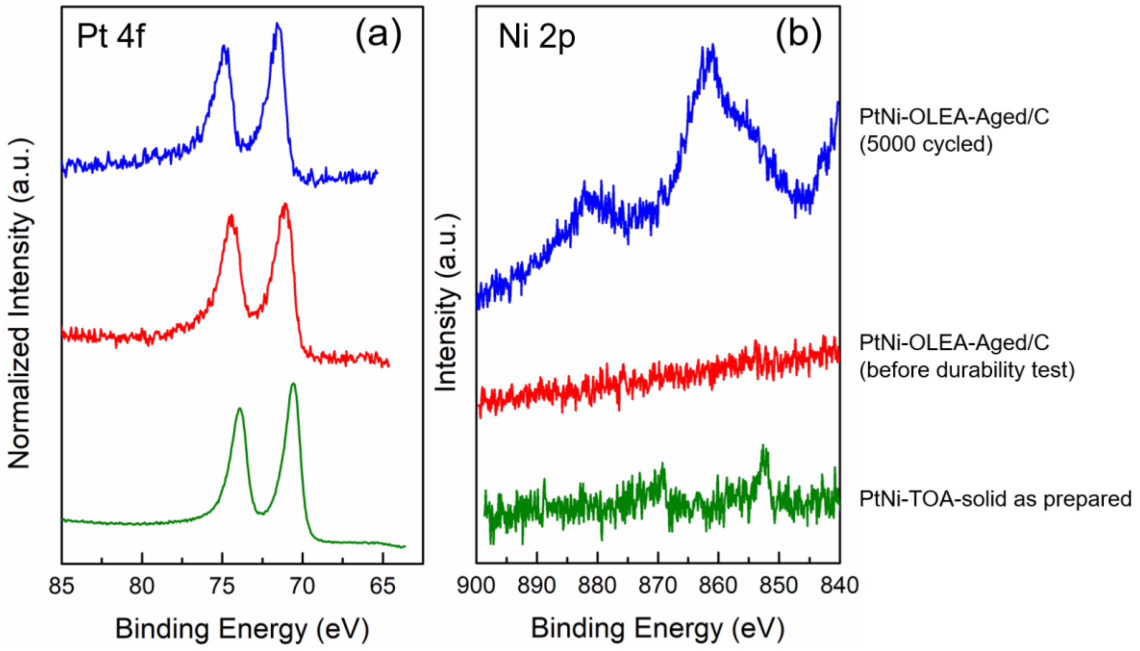

Figure S8. Normalised Pt 4f (**a**) and Ni 2p (**b**) high-resolution XPS spectra for PtNi nanoparticles: as-prepared, Aged/C and Aged/C after 5000 cycles. Results for TOA and OLEA routes are similar. That the XPS Ni peak is much weaker than the Pt peak in the as-prepared sample, which has an average composition of Pt_1Ni_2, is suggestive of a core/shell structure with an outer Pt rich shell. In this case, the shell thickness should be similar to the information depth of the XPS measurement, defined as 3* Inelastic Mean Free Path (IMFP) of electrons from the Ni 2p peak in Pt metal. The IMFP of 630 eV photoelectrons in Pt metal using the TPP formula[19] at 0.95 nm is calculated to be 2.85 nm, which indicates the lower limit for the Pt shell thickness is around 2.85 nm, consistent with the STEM-HAADF analysis of a shell thickness of 3-4 atomic layers (Figure 1(h-m)).

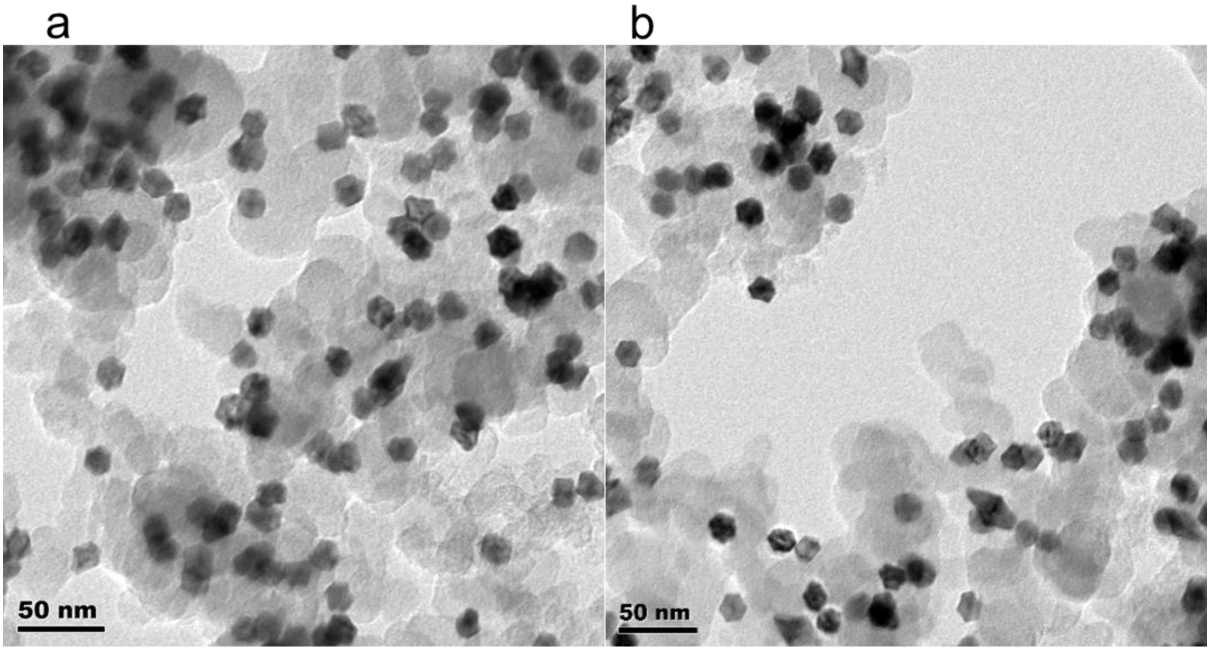

Figure S9. BF-TEM images of (**a**) PtNi-OLEA-Solid/C and (**b**) PtNi-TOA-Solid/C nanoparticles.

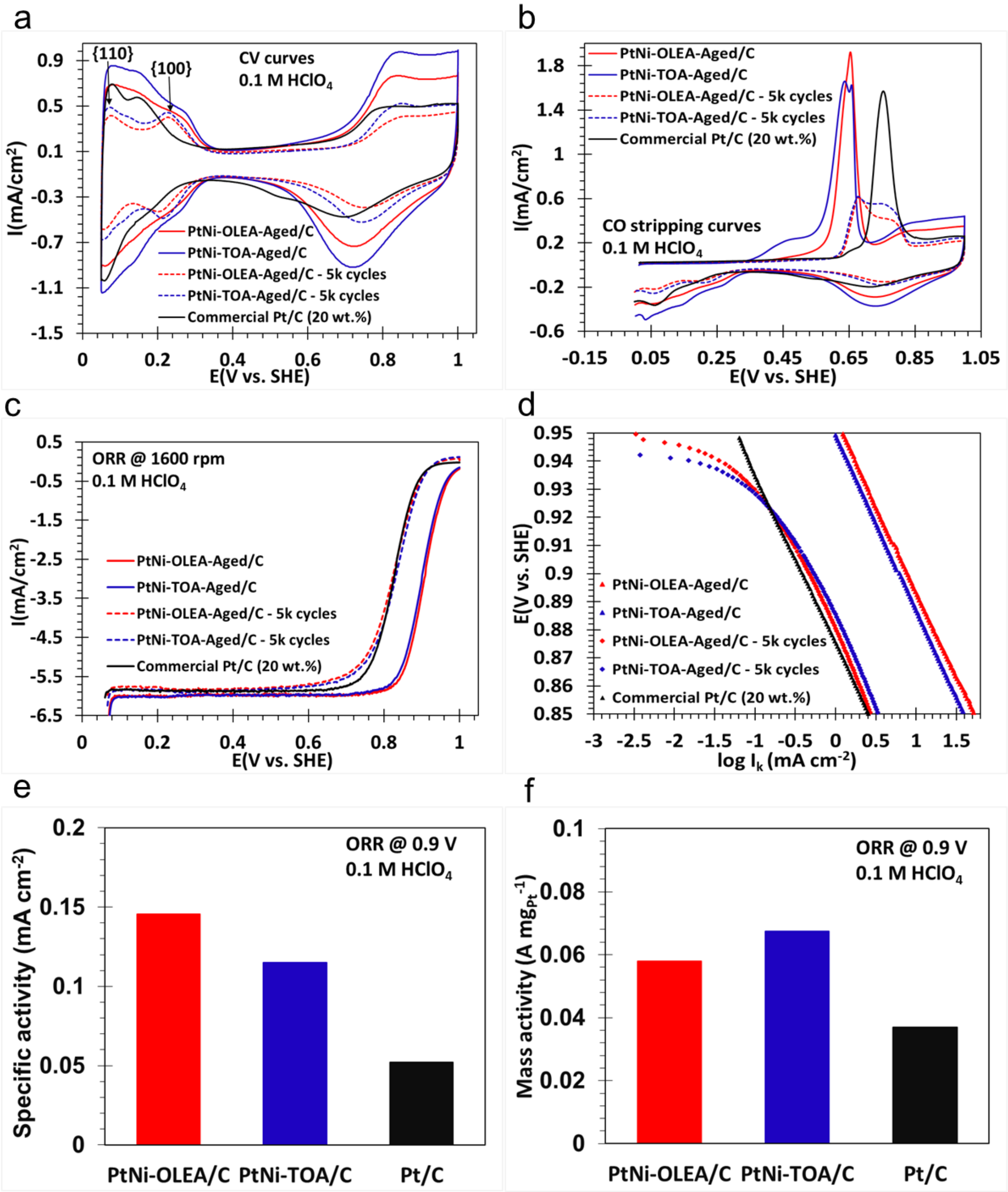

Figure S10. (**a**) Cyclic voltammograms of binary PtNi-OLEA-Aged/C (red), PtNi-TOA-Aged/C (blue) and commercial Pt/C (black) electrocatalysts showing the evolution of two peaks (indicated) indexed to {100} and {110} planes, (**b**) CO-stripping voltammetry curves, (**c**) ORR polarization curves, (**d**) the corresponding Tafel plots, (**e**) intrinsic area-specific activities and (**f**) mass-specific activities at +0.9 V (vs SHE), post 5000 electrochemical cycles.

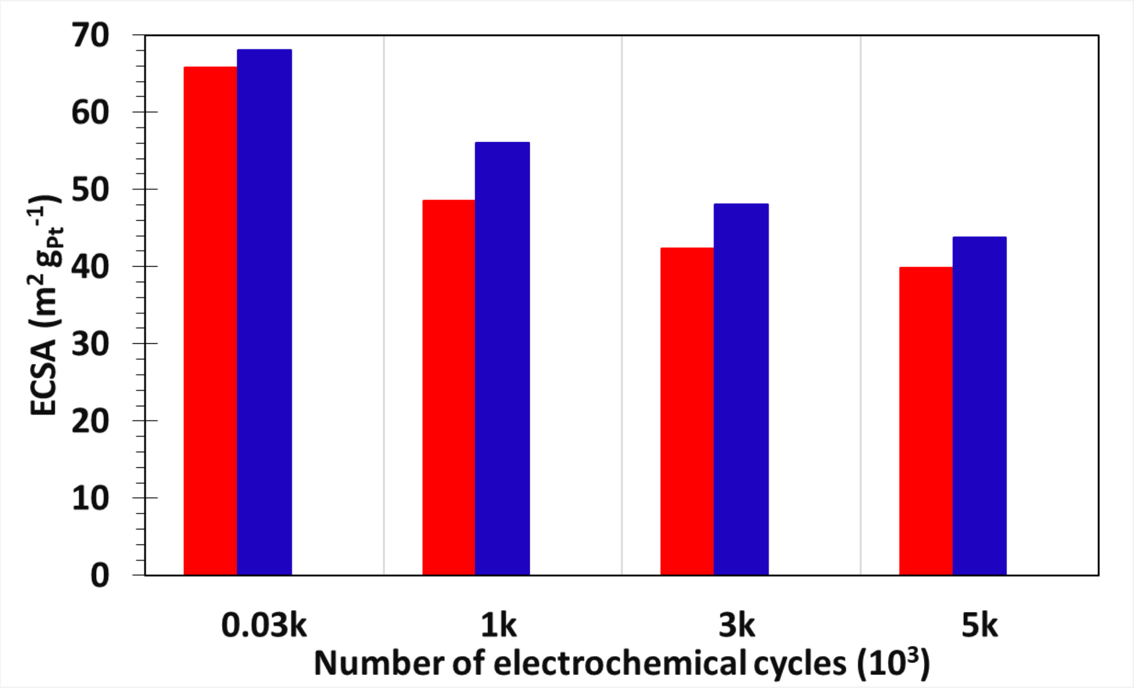

Figure S11. ECSA decay as a function of the continual potential cycling (up to 5000 cycles) of PtNi-OLEA-Aged/C (red) and PtNi-TOA-Aged/C (blue) binary nanoparticles.

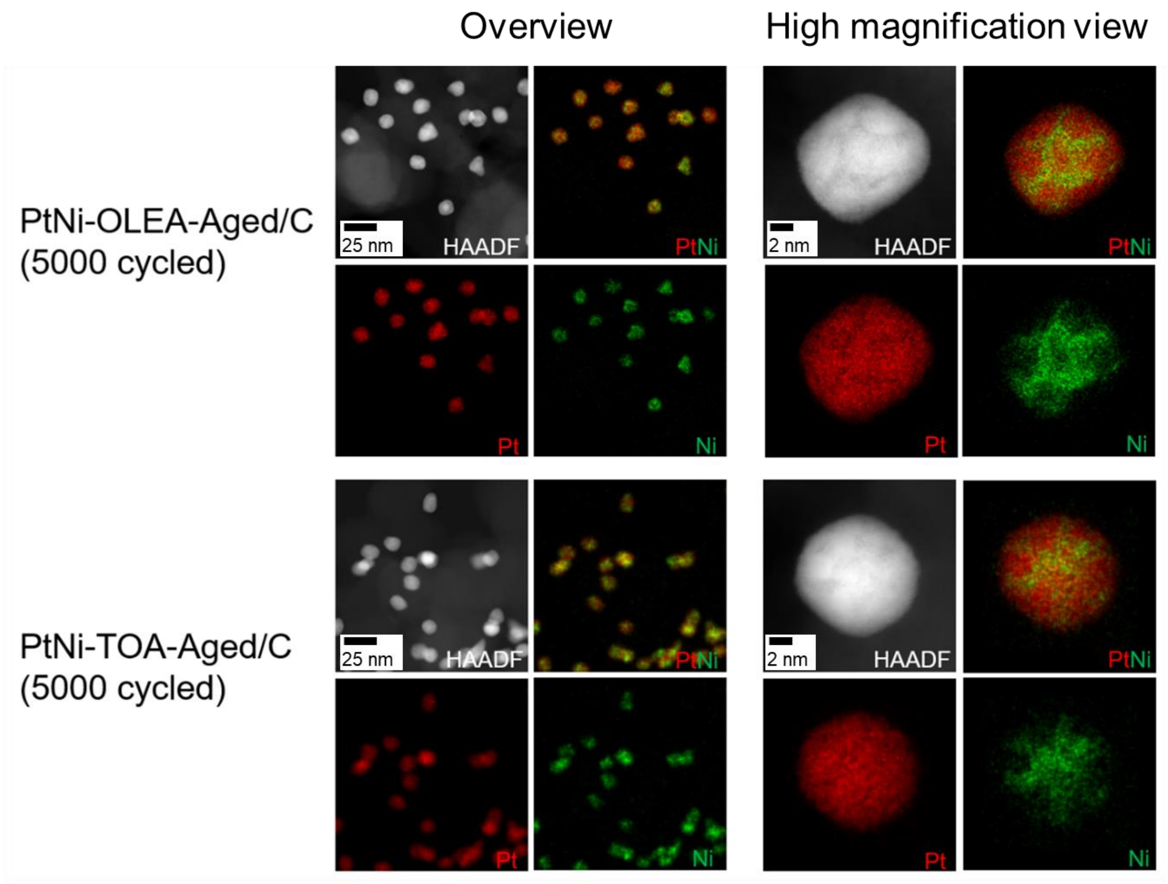

Figure S12. HAADF STEM and STEM EDXS elemental maps of PtNi-OLEA-Aged/C and PtNi-TOA-Aged/C nanoparticles after 5000 continual potential cycling.

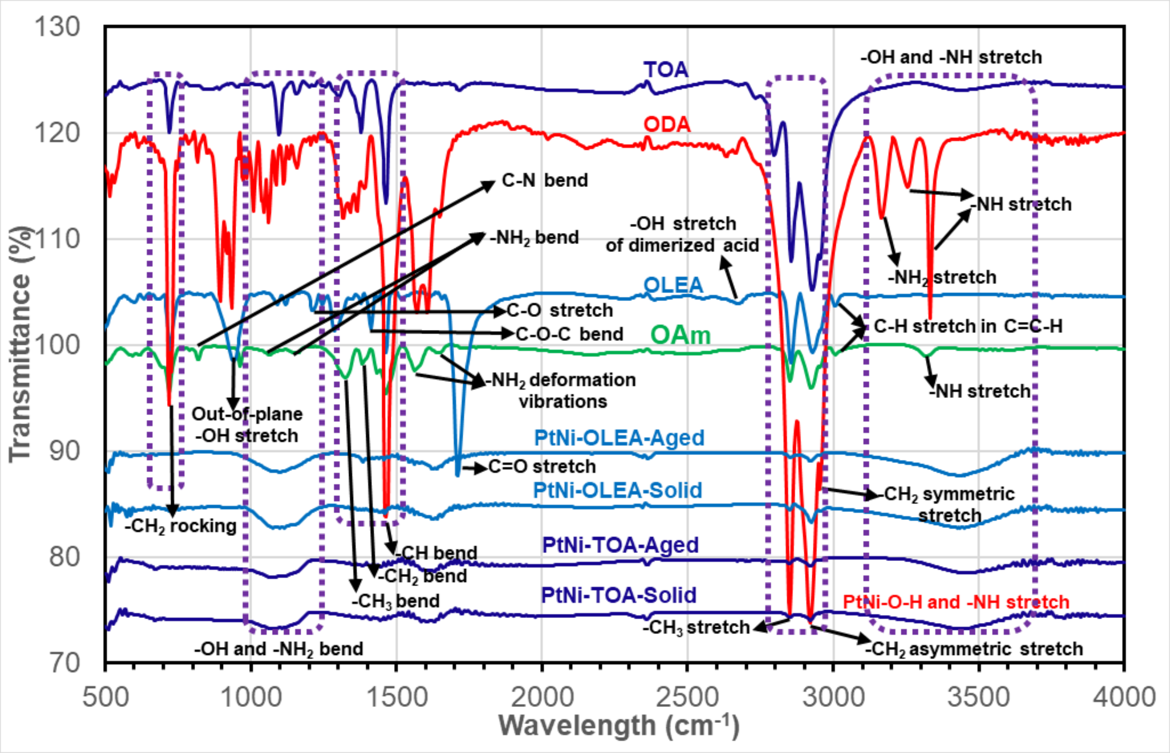

Figure S13. Fourier transform infrared measurements of all surfactants (TOA, ODA, OLEA, OAm) and all samples (PtNi-OLEA-Solid, PtNi-OLEA-Aged, PtNi-TOA-Solid, PtNi-TOA-Aged).

Table S1. Measured and calculated d-spacings and lattice parameters. Note that for bulk fcc Pt and Ni the lattice constants are 0.3912 nm and 0.3499 nm,[20] respectively.

Sample	XRD			HAADF-STEM		TEM Diffraction	
	2θ diffraction peaks (°)	Measured d-spacing (nm)	Calculated lattice parameter (nm)	Measured d-spacing (nm)	Calculated lattice parameter (nm)	Measured d-spacing (nm)	Calculated lattice parameter (nm)
PtNi-OLEA-Solid	41.534	0.217 {111}	0.376	0.22 ± 0.01 {111}	0.38 ± 0.02	-	-
PtNi-OLEA-Aged	40.693	0.222 {111}	0.384	0.19 ± 0.01 {200}	0.38 ± 0.02	-	-
PtNi-TOA-Solid	41.841	0.216 {111}	0.374	0.22 ± 0.01 {111}	0.38 ± 0.02	0.215 ± 0.005 {111}<br>0.185 ± 0.005 {200}	0.372 ± 0.009<br>0.370 ± 0.010
PtNi-TOA-Aged	41.023	0.220 {111}	0.381	0.22 ± 0.01 {111}	0.38 ± 0.02	0.222 ± 0.005 {111}<br>0.190 ± 0.005 {200}	0.385 ± 0.009<br>0.381 ± 0.010

Table S2. Comparisons of the $ECSA_{CO}$, $ECSA_{Hupd}$, mass-specific and area-specific activities at 0.9 V and $ECSA_{CO}/ECSA_{Hupd}$ of two binary electrocatalysts before and after extended durability measurements.

Electrocatalysts	ECSA (m^2/g_{Pt}) (CV)	ECSA (m^2/g_{Pt}) (CV) loss (%)	ECSA (m^2/g_{Pt}) (CO stripping)	ECSA (m^2/g_{Pt}) (CO stripping) loss (%)	Mass activity (A/mg_{Pt})	Mass activity loss (%)	Specific activity (mA/cm^2_{Pt})	Specific activity loss (%)
PtNi-OLEA-Solid/C	0.1k cycles: 56.2	-	-	-	0.33	-	0.59	-
PtNi-TOA-Solid/C	0.1k cycles: 59.4	-	-	-	0.30	-	0.51	-
PtNi-OLEA-Aged/C	0.03k cycles: 65.7	39.4	0.03k cycles: 71.7	39.5	0.91	93.7	1.39	90
	5k cycles: 39.8		5k cycles: 43.4		0.06		0.15	
PtNi-TOA-Aged/C	0.03k cycles: 68.1	35.7	0.03k cycles: 74.7	32.9	0.75	91.0	1.10	90
	5k cycles: 43.8		5k cycles: 50.1		0.07		0.12	
Pt/C	0.1k cycles: 71.0	-	0.1k cycles: 87.2	-	0.037	-	0.052	-

ECSA (CO_{ad})/ECSA (H_{upd})	PtNi-OLEA-Aged/C	PtNi-TOA-Aged/C
0.03k cycles	1.10	1.10
5k cycles	1.10	1.14

Table S3. Comparison of the ORR activities over Pt alloy catalysts in recent studies

Catalyst	Particle size (nm)	Metal loading	Electrochemical surface area ($m^2g_{Pt}^{-1}$)	Area-specific activities ($mA\cdot cm^{-2}$)	Mass-specific activities ($A\cdot mg_{Pt}^{-1}$)
$Pt_{1.5}Ni$ octahedral/C[21]	5.8±1.5	20.4wt%	48.3 ^{a}	3.99^{a}	1.96
PtNi octahedra/C[22]	7.98±0.38	29.4wt%	45.0	/	~1.8
PtNi octahedra/C[23]	9	~25wt%	45.0 ^{b}	10.1^{b}	3.30
PtNi octahedral/C[24]	9.5±0.8	/	50.0 ^{b}	3.14^{b}	1.45
Pt_3Ni octahedra/C[25]	10	20wt%	/	1.26	0.44
Pt_2Ni octahedra/C[26]	9.5±0.5	/	/	/	2.0
PtNi octahedra/C[27]	12±0.8	/	/	~3.8^{b}	1.70
Nanoporous NiPt/C[28]	15	/	41.0 ^{a}	3.34^{a}	1.32
PtNi cubes/C[29]	8-9	/	/	3.0^{a}	0.68
Pt_3Ni octahedra/C[30]	~4	/	66.6 ^{a}	2.7^{a}	1.80
Pt_3Ni octahedra/C[31]	11	/	/	2.8^{a}	0.11
PtNi octahedra/C[32]	7.10 ± 1.1	/	38.1 ^{a}	2.42^{a}	0.92
PtNi hexapods/C[33]	10-20	/	47.2	/	0.85
Pt_3Ni nanoframe/C[34]	~20	~20wt%	/	1.52^{b*}	5.7
Jagged PtNi nanowires/C[35]	/	/	118.0 ^{a}	11.5^{a}	13.6
$PtCo_3$ nanoparticles/C[36]	8.9 ± 2.0	/	60.0	2.24	1.14
PtCo nanocubes/graphene[14]	5.5 ± 0.5	/	61.3	~0.5	0.95
$Pt_{68}Cu_{32}$ nanoparticles[37]	4.84± 0.07	/	12.1 ^{a}	2.85^{a}	0.34
PtCu nanoframes/C[38]	~40	~20wt%	66.2 ^{a}	1.24^{a}	0.82
**Pt/Ni-OLEA-Aged/C (This work)**	**17.9±1.5**	**~20wt%**	**65.7 ^{a}**	**1.39^{a}**	**0.91**
**Pt/Ni-TOA-Aged/C (This work)**	**16.6±1.5**	**~20wt%**	**68.1 ^{a}**	**1.10^{a}**	**0.75**

Both area and mass specific activities are measured at 0.90 V in 0.1M $HClO_4$ electrolyte.
^{a}Normalized to the surface areas derived from the H adsorption charges. ^{b}Normalized to the surface areas derived from the CO stripping charges. *Activities are measured at 0.95 V in 0.1M $HClO_4$ electrolyte.

References

(1) Wang, Y. C.; Slater, T. J. A.; Leteba, G. M.; Roseman, A. M.; Race, C. P.; Young, N. P.; Kirkland, A. I.; Lang, C. I.; Haigh, S. J. Imaging Three-Dimensional Elemental Inhomogeneity in Pt-Ni Nanoparticles Using Spectroscopic Single Particle Reconstruction. *Nano Lett.* **2019,** *19* (2), 732-738.

(2) Koch, C. Determination of Core Structure Periodicity and Point Defect Density along Dislocations. PhD thesis, Arizona State University, **2002**.

(3) Nord, M.; Vullum, P. E.; MacLaren, I.; Tybell, T.; Holmestad, R. Atomap: A New Software Tool for the Automated Analysis of Atomic Resolution Images Using Two-dimensional Gaussian Fitting. *Adv. Struct. Chem. Imaging* **2017,** *3* (1), 9.

(4) Lee, S.; Kim, H. J.; Choi, S. M.; Seo, M. H.; Kim, W. B. The Promotional Effect of Ni on Bimetallic PtNi/C Catalysts for Glycerol Electrooxidation. *Appl. Catal., A* **2012,** *429-430*, 39-47.

(5) Choi, E.; Oh, S.; Choi, M. Charge Transfer in Ni_xPt_{1-x} Alloys Studied by X-ray Photoelectron Spectroscopy. *Phys. Rev. B* **1991,** *43* (8), 6360-6368.

(6) Ralph, T. R.; Hards, G. A.; Keating, J. E.; Campbell, S. A.; Wilkinson, D. P.; Davis, M.; St-Pierre, J.; Johnson, M. C. Low Cost Electrodes for Proton Exchange Membrane Fuel Cells: Performance in Single Cells and Ballard Stacks. *J. Electrochem. Soc.* **1997,** *144*, 3845.

(7) Shui, J.; Chen, C.; Li, J. C. M. Evolution of Nanoporous Pt-Fe Alloy Nanowires by Dealloying and their Catalytic Property for Oxygen Reduction Reaction. *Adv. Funct. Mater.* **2011,** *21* (17), 3357-3362.

(8) Maillard, F.; Schreier, S.; Hanzlik, M.; Savinova, E. R.; Weinkauf, S.; Stimming, U. Influence of Particle Agglomeration on the Catalytic Activity of Carbon-supported Pt Nanoparticles in CO Monolayer Oxidation. *Phys. Chem. Chem. Phys.* **2005,** *7* (2), 385-393.

(9) Gasteiger, H. A.; Kocha, S. S.; Sompalli, B.; Wagner, F. T. Activity Benchmarks and Requirements for Pt, Pt-alloy, and Non-Pt Oxygen Reduction Catalysts for PEMFCs. *Appl. Catal. B* **2005,** *56* (1-2), 9-35.

(10) Huang, X.; Zhu, E.; Chen, Y.; Li, Y.; Chiu, C. Y.; Xu, Y.; Lin, Z.; Duan, X.; Huang, Y. A Facile Strategy to Pt_3Ni Nanocrystals with Highly Porous Features as an Enhanced Oxygen Reduction Reaction Catalyst. *Adv. Mater.* **2013,** *25* (21), 2974-2979.

(11) Heinze, J. Allen J. Bard and Larry F. Faulkner: Electrochemical Methods - Fundamentals and Applications. Wiley, New York 1980, 718 + XVIII S., Preis: £ 14.70. *Berichte der Bunsengesellschaft für Phys. Chemie* **1981**, *85*, 1085 – 1086.

(12) Paulus, U. A.; Schmidt, T. J.; Gasteiger, H. A.; Behm, R. J. Oxygen Reduction on a High-surface Area Pt/Vulcan Carbon Catalyst: a Thin-film Rotating Ring-disk Electrode Study. *J. Electroanal. Chem.* **2001,** *495*, 134 – 145.

(13) Garsany, Y.; Baturina, O. A.; Swider-Lyons, K. E.; Kocha, S. S. Experimental Methods for Quantifying the Activity of Platinum Electrocatalysts for the Oxygen Reduction Reaction. *Anal. Chem.* **2010,** *82*, 6321.

(14) He, C.; Zhang, S.; Tao, J.; Shen, P. One-step Solid State Synthesis of PtCo Nanocubes/graphene Nanocomposites as Advanced Oxygen Reduction Reaction Electrocatalysts. *J. Catal.* **2018,** *362*, 85-93.

(15) Stamenkovic, V. R.; Mun, B. S.; Arenz, M.; Mayrhofer, K. J.; Lucas, C. A.; Wang, G.; Ross, P. N.; Markovic, N. M. Trends in Electrocatalysis on Extended and Nanoscale Pt-bimetallic Alloy Surfaces. *Nat. Mater.* **2007,** *6* (3), 241-247.

(16) Yang, H. T.; Su, Y. K.; Shen, C. M.; Yang, T. Z.; Gao, H. J. Synthesis and Magnetic Properties of ε-cobalt Nanoparticles. *Surf. Interface Anal.* **2004,** *36* (2), 155-160.

(17) Erley, W.; Hemminger, J. C. Spectroscopic Identification of an HCNH Species on Pt(111). *Surf. Sci.* **1994,** *316*, L1025-L1030.

(18) Kita, H.; Ye, S.; Aramata, A.; Furuya, N. Adsorption of Hydrogen on Platinum Single Crystal Electrodes in Acid and Alkali Solutions. *J. Electroanal. Chem. Interfacial Electrochem.* **1990,** *295*, 317–331.

(19) Tanuma, S.; Powell, C. J.; Penn, D. R. Calculations of Electron Inelastic Mean Free Paths. *Surf. Interface Anal.* **1991,** *17*, 911-926.

(20) Davey, W. P. Precision Measurements of the Lattice Constants of Twelve Common Metals. *Phys. Rev.* **1925,** *25* (6), 753-761.
(21) Zhang, C.; Hwang, S. Y.; Trout, A.; Peng, Z. Solid-state Chemistry-enabled Scalable Production of Octahedral Pt-Ni Alloy Electrocatalyst for Oxygen Reduction Reaction. *J. Am. Chem. Soc.* **2014,** *136* (22), 7805-7808.
(22) Kühl, S.; Gocyla, M.; Heyen, H.; Selve, S.; Heggen, M.; Dunin-Borkowski, R. E.; Strasser, P. Concave Curvature Facets Benefit Oxygen Electroreduction Catalysis on Octahedral Shaped PtNi Nanocatalysts. *J. Mater. Chem. A* **2019,** *7* (3), 1149-1159.
(23) Choi, S. I.; Xie, S.; Shao, M.; Odell, J. H.; Lu, N.; Peng, H. C.; Protsailo, L.; Guerrero, S.; Park, J.; Xia, X.; Wang, J.; Kim, M. J.; Xia, Y. Synthesis and Characterization of 9 nm Pt-Ni Octahedra with a Record High Activity of 3.3 A/mg(Pt) for the Oxygen Reduction Reaction. *Nano Lett.* **2013,** *13* (7), 3420-3425.
(24) Cui, C.; Gan, L.; Li, H. H.; Yu, S. H.; Heggen, M.; Strasser, P. Octahedral PtNi Nanoparticle Catalysts: Exceptional Oxygen Reduction Activity by Tuning the Alloy Particle Surface Composition. *Nano Lett.* **2012,** *12* (11), 5885-5889.
(25) Wu, J.; Gross, A.; Yang, H. Shape and Composition-controlled Platinum Alloy Nanocrystals Using Carbon Monoxide as Reducing Agent. *Nano Lett.* **2011,** *11* (2), 798-802.
(26) Chang, Q.; Xu, Y.; Duan, Z.; Xiao, F.; Fu, F.; Hong, Y.; Kim, J.; Choi, S. I.; Su, D.; Shao, M. Structural Evolution of Sub-10 nm Octahedral Platinum-Nickel Bimetallic Nanocrystals. *Nano Lett.* **2017,** *17* (6), 3926-3931.
(27) Cui, C.; Gan, L.; Heggen, M.; Rudi, S.; Strasser, P. Compositional Segregation in Shaped Pt Alloy Nanoparticles and their Structural Behaviour during Electrocatalysis. *Nat. Mater.* **2013,** *12* (8), 765-771.
(28) Joshua, S.; Kenneth, L.; Jonah, E. Oxygen Reduction Reaction Performance of [MTBD][beti]-Encapsulated Nanoporous NiPt Alloy Nanoparticles. *Adv. Funct. Mater.* **2013,** *23* (44), 5494-5501.
(29) Carpenter, M. K.; Moylan, T. E.; Kukreja, R. S.; Atwan, M. H.; Tessema, M. M. Solvothermal Synthesis of Platinum Alloy Nanoparticles for Oxygen Reduction Electrocatalysis. *J. Am. Chem. Soc.* **2012,** *134* (20), 8535-8542.
(30) Huang, X.; Zhao, Z.; Cao, L.; Chen, Y.; Zhu, E.; Lin, Z.; Li, M.; Yan, A.; Zettl, A.; Wang, Y. M.; Duan, X.; Mueller, T.; Huang, Y. High-performance Transition Metal－doped Pt_3Ni Octahedra for Oxygen Reduction Reaction. *Science* **2015,** *348*, 1230.
(31) Zhang, J.; Yang, H.; Fang, J.; Zou, S. Synthesis and Oxygen Reduction Activity of Shapecontrolled Pt_3Ni Nanopolyhedra. *Nano Lett.* **2010,** *10* (2), 638-644.
(32) Choi, J.; Jang, J.; Roh, C.; Yang, S.; Kim, J.; Lim, J.; Yoo, S.; Lee, H. Gram-scale Synthesis of Highly Active and Durable Octahedral PtNi Nanoparticle Catalysts for Proton Exchange Membrane Fuel Cell. *Appl. Catal. B* **2018,** *225*, 530-537.
(33) Song, X.; Luo, S.; Fan, X.; Tang, M.; Zhao, X.; Chen, W.; Yang, Q.; Quan, Z. Controlled Synthesis of PtNi Hexapods for Enhanced Oxygen Reduction Reaction. *Front. Chem.* **2018,** *6*, 468.
(34) Chen, C.; Kang, Y.; Huo, Z.; Zhu, Z.; Huang, W.; Xin, H. L.; Snyder, J. D.; Li, D.; Herron, J. A.; Mavrikakis, M.; Chi, M.; More, K. L.; Li, Y.; Markovic, N. M.; Somorjai, G. A.; Yang, P.; Stamenkovic, V. R. Highly Crystalline Multimetallic Nanoframes with Three-Dimensional Electrocatalytic Surfaces. *Science* **2014,** *343*, 1339.
(35) Li, M.; Zhao, Z.; Cheng, T.; Fortunelli, A.; Chen, C.; Yu, R.; Zhang, Q.; Gu, L.; Merinov, B. V.; Lin, Z.; Zhu, E.; Yu, T.; Jia, Q.; Guo, J.; Zhang, L.; Goddard III, W. A.; Huang, Y.; Duan, X. Ultrafine Jagged Platinum Nanowires Enable Ultrahigh Mass Activity for the Oxygen Reduction Reaction. *Science* **2016,** *354*, 1414.
(36) Lee, J. D.; Jishkariani, D.; Zhao, Y.; Najmr, S.; Rosen, D.; Kikkawa, J. M.; Stach, E. A.; Murray, C. B. Tuning the Electrocatalytic Oxygen Reduction Reaction Activity of Pt-Co Nanocrystals by Cobalt Concentration with Atomic-Scale Understanding. *ACS Appl. Mater. Interfaces* **2019,** *11* (30), 26789-26797.
(37) Liu, T.; Wang, K.; Yuan, Q.; Shen, Z.; Wang, Y.; Zhang, Q.; Wang, X. Monodispersed Sub-5.0 nm PtCu Nanoalloys as Enhanced Bifunctional Electrocatalysts for Oxygen Reduction Reaction and Ethanol Oxidation Reaction. *Nanoscale* **2017,** *9* (9), 2963-2968.

(38) Gong, M.; Xiao, D.; Deng, Z.; Zhang, R.; Xia, W.; Zhao, T.; Liu, X.; Shen, T.; Hu, Y.; Lu, Y.; Zhao, X.; Xin, H.; Wang, D. Structure Evolution of PtCu Nanoframes from Disordered to Ordered for the Oxygen Reduction Reaction. *Appl. Catal. B* **2021,** *282*, 119617.
